# Gene expression during bacterivorous growth of a widespread marine heterotrophic flagellate

**DOI:** 10.1038/s41396-020-00770-4

**Published:** 2020-09-12

**Authors:** Ramon Massana, Aurelie Labarre, David López-Escardó, Aleix Obiol, François Bucchini, Thomas Hackl, Matthias G. Fischer, Klaas Vandepoele, Denis V. Tikhonenkov, Filip Husnik, Patrick J. Keeling

**Affiliations:** 1 Institut de Ciències del Mar (CSIC), Passeig Marítim de la Barceloneta 37-49, ES-08003 Barcelona, Catalonia Spain; 2 Department of Plant Systems Biology, VIB, B-9052 Ghent, Belgium; 3 Department of Plant Biotechnology and Bioinformatics, Ghent University, B-9052 Ghent, Belgium; 4 Max Planck Institute for Medical Research, 69120 Heidelberg, Germany; 5 Papanin Institute for Biology of Inland Waters, Russian Academy of Sciences, Borok, 152742 Russia; 6 University of British Columbia, Vancouver, BC V6T 1Z4 Canada

**Keywords:** Water microbiology, Microbial biooceanography

## Abstract

Phagocytosis is a fundamental process in marine ecosystems by which prey organisms are consumed and their biomass incorporated in food webs or remineralized. However, studies searching for the genes underlying this key ecological process in free-living phagocytizing protists are still scarce, in part due to the lack of appropriate ecological models. Our reanalysis of recent molecular datasets revealed that the cultured heterotrophic flagellate *Cafeteria burkhardae* is widespread in the global oceans, which prompted us to design a transcriptomics study with this species, grown with the cultured flavobacterium *Dokdonia* sp. We compared the gene expression between exponential and stationary phases, which were complemented with three starvation by dilution phases that appeared as intermediate states. We found distinct expression profiles in each condition and identified 2056 differentially expressed genes between exponential and stationary samples. Upregulated genes at the exponential phase were related to DNA duplication, transcription and translational machinery, protein remodeling, respiration and phagocytosis, whereas upregulated genes in the stationary phase were involved in signal transduction, cell adhesion, and lipid metabolism. We identified a few highly expressed phagocytosis genes, like peptidases and proton pumps, which could be used to target this ecologically relevant process in marine ecosystems.

## Introduction

Eukaryotic microbes (protists) include a diverse collection of unicellular organisms that are involved in crucial food web processes such as primary production, predation, and parasitism [1, 2]. A particular functional group, referred as heterotrophic flagellates, are known to be primary agents of bacterivory. As such, they keep bacterial abundances in check, direct bacterial production to higher trophic levels, and release inorganic nutrients that sustain regenerated primary production [3, 4]. For years, the abundance, distribution, and activity of heterotrophic flagellates was studied as a group property and their diversity addressed by morphological and culturing approaches [5, 6]. The advent of molecular tools revealed many uncultured and undescribed species [7, 8], highlighted a prevalent culturing bias, and suggested many of the isolated species were rare in nature and perhaps poor models for more dominant ones [9]. Little work has been done linking physiological studies of cultured heterotrophic flagellates with the genes responsible for ecologically relevant processes, despite the great promise of transcriptomics to provide new insights into the ecology of eukaryotic species [10].

Heterotrophic flagellates feed on bacteria through phagocytosis, the engulfment and digestion of a prey cell in a food vacuole. Phagocytosis is an ancient trait that marked the origin of eukaryotic cells [11] and allowed critical evolutionary innovations [12, 13]. It is a complex process involving hundreds of proteins operating in consecutive steps: sensing and motility, prey recognition, cytoskeleton remodeling for food vacuole formation, vacuole maturation, and acidic enzymatic digestion. Given its importance in immunity [14], phagocytosis has been mostly investigated at the cellular and molecular level in metazoan immune cells [15, 16], where identified genes have been placed in functional maps [17]. The few studies done with free-living protists, like ciliates and amoebozoans [18, 19], indicate that the basic machinery for phagocytosis and many of the genes involved are evolutionarily conserved [20]. However, these studies do not provide a detailed model of how gene expression changes during phagocytic growth, and this could be readily studied by differential expression (DE) analyses of cells actively preying versus starved ones. This experiment has rarely been performed [21, 22], due to the lack of cultured ecological models.

We studied the bicosoecid *Cafeteria burkhardae*, an efficient suspension feeder that preys on bacteria by creating a current with its anterior flagellum. Although the used strain E4-10 was named *C. roenbergensis*, a recent paper that sequenced the 18S rDNA of the type species *C. roenbergensis* [23] showed that both strains had different 18S rDNA, which led to the description of *C. burkhardae* [24]. *C. burkhardae* strain E4-10 was used in the MMETSP transcriptome initiative [25] and its high-quality draft genome has been recently released [26]. Moreover, the strains easily cultured from seawater [5] and often used in growth and grazing experiments [27, 28] also correspond to *C. burkhardae* [24]. Previous studies suggested this species was a minor member of marine heterotrophic flagellates [29], but we describe here more extensive molecular surveys that reveal a widespread distribution. We grew *C. burkhardae* in batch cultures with a known bacterium and collected transcriptomic samples at the exponential and stationary phases, together with additional states where the cells were starved by dilution. DE analysis identified genes correlated with exponential growth, when cells were feeding, converting bacterial food to biomass and dividing. Some of these genes, particularly those that were highly expressed, are promising targets for future exploration of phagocytosis in marine ecosystems.

## Material and methods

### *C. burkhardae* in the Malaspina dataset

Marine microbes (0.2–3 µm size fraction) were collected during the Malaspina expedition in 120 stations at surface and in 13 profiles of 7 depths from surface to the bathypelagic zone. Eukaryotic diversity was assessed by sequencing the V4 18S rDNA region. Details of sample collection, nucleic acid extraction, V4 amplification, and Illumina sequencing are presented elsewhere for surface data [30] and vertical profiles [31]. Here, we processed the reads using DADA2 [32] with parameters *truncLen* 240,210 and *maxEE* 6,8 and identified the ASV (Amplicon Sequence Variant) corresponding to *C. burkhardae*. Its relative abundance was calculated against the number of reads per sample after removal of metazoan and plant reads. Metagenomes of the same size fraction in vertical profiles were generated from the same cruise [33] and used in BLAST [34] fragment recruitment analysis against the *C. burkhardae* genome [24]. Direct cell counts were performed in 13 surface samples by FISH as explained before [29, 35].

### Growth of *C. burkhardae* on *Dokdonia* sp.

The flavobacterium *Dokdonia* sp. MED134 was isolated on Zobell agar plates from the Blanes Bay Microbial Observatory [36]. To prepare cell concentrates, a colony was inoculated in 50 mL of Zobell medium and incubated at 22 °C for 3 days. Cells were collected by centrifugation (4500 rpm for 15 min), resuspended in sterile seawater (filtered by 0.2 µm and autoclaved), centrifuged again, resuspended in 100 mL of sterile seawater, and kept at 4 °C for 1 week. To calculate the cell abundance of the concentrate, one aliquot was fixed with ice-cold glutaraldehyde (1% final concentration), stained with DAPI, and filtered on a 0.2 µm pore-size polycarbonate filter. Filters were mounted on a slide and counts were performed by epifluorescence microscopy by exciting with UV radiation [37].

*C. burkhardae* strain E4-10 was isolated in 1989 [38] and maintained on a rice grain with artificial seawater. The culture was acclimated to grow on *Dokdonia* MED134 as prey in two steps. First 0.1 mL of the culture was inoculated in a flask with 20 mL of sterile seawater and 10^8^ bacteria mL^−1^ for 5 days. Second, 1 mL of this culture was inoculated to 400 mL of sterile seawater and 2.4 × 10^7^ bacteria mL^−1^ for 1 week. Flagellate growth was inspected by light microscopy through the culture flasks. Incubations were done at 22 °C on the lab bench.

### Batch cultures, dilution event, and RNA extraction and sequencing

Three batch cultures were prepared with 400 mL of sterile seawater, *Dokdonia* MED134 at 2.5 × 10^7^ cells mL^−1^, and 1 mL of *C. burkhardae* from the last acclimation bottle. Three milliliters aliquots were fixed with glutaraldehyde to count, just after sampling, the abundance of flagellates and bacteria by epifluorescence microscopy. Flagellate growth rates were calculated as the slope of the linear part of logarithmic cell numbers versus time. Grazing rates were calculated using growth rates, the slope of the logarithmic decrease of bacteria, and the geometric mean of flagellates and bacteria abundances using the formulas of Frost [39] and Heinbokel [40]. Growth efficiency was calculated from growth and grazing rates and the estimated carbon per cell of both species obtained from cell sizes measured at the microscope [41].

Samples for transcriptomics were taken in triplicates from the last acclimation bottle (Inoculum), and in duplicates in the three bottles at the exponential (day 2.3) and stationary (day 3.7) phases. Cells were collected in microfiltration units of 0.8 µm pore size (Vivaclear MINI 0.8 µm PES, Sartorius, Göttingen, Germany). For each sample, four units were filled with 0.5 mL of culture, spun down for 30 s at 1000 rpm, and the step repeated until processing 10 mL. Next, 100 µL of lysis buffer from the RNAqueous-Micro kit (Thermo Fisher Scientific, Waltham, Massachusetts, US) were added to each unit, vortexed, left for 1 min, and the lysate was spun down at 13,000 rpm for 30 s. The four cell lysates from the same sample were combined and the RNA was extracted following the kit’s protocol. Genomic DNA was removed with DNase I. RNA quantity and purity was assessed with a NanoDrop 1000 Spectrophotometer (Thermo Fisher Scientific) and the RNA extracts were kept at −80 °C.

During the exponential phase, three dilutions (10 mL of culture in 190 mL sterile seawater) were prepared from each batch culture, and they were processed after 0.4, 1.4, and 3.3 days for cell counts (5 mL) and RNA extraction (195 mL). As these large volumes prevented the use of microfiltration units, cell collection was done on 47 mm polycarbonate filters of 0.8 µm pore size. Filters were cut in four pieces, submerged in 1 mL of lysis buffer, vortexed, and left for 30 s. The lysate was recovered and the RNA was extracted as before.

Polyadenylated RNA transcripts were converted into cDNA following the Smart-seq2 protocol [42] designed for very low RNA amounts. In brief, Oligo-dT_30_VN primers annealed to all mRNAs containing a poly(A) tail, then reverse transcription and template-switching was done, followed by 9-cycles of PCR amplification using IS PCR oligos linked at the two ends of the cDNA molecules [42]. Amplified cDNA was purified and quantified with a Qubit fluorometer (Thermo Fisher Scientific). The complete set of 24 cDNA samples (15 µL at 2–4 ng L^−1^) was sent to the Sequencing + Bioinformatics Consortium at UBC and, based on the BioAnalyzer results (Agilent, Santa Clara, California, US), 21 samples were chosen for sequencing (Table S1). Illumina Nextera XT libraries with a dual index were prepared and pooled on a single lane of a NextSeq Illumina sequencer yielding, on average, 14.1 million 150 bp pair-ended reads per sample (Table S1). Raw reads have been deposited in ENA under the accession number PRJEB36247.

### Transcriptome assembly, functional annotation, and DE analysis

Quality trimming of Illumina reads was done using Trimmomatic 0.33 [43] with parameters set to crop:149 slidingwindow:6:25 minlen:50. This removed about one third of the reads per sample (Table S1). High-quality reads were mapped with Bowtie2 [44] towards the genome of *Dokdonia* MED134 (3.3 Mb; CP009301) and the *C. burkhardae* rDNA operon (5800 bp; extracted from a genome contig with the 18S rDNA [KY886365] and the 28 S rDNA [FJ032656]). We used Bowtie2 in the sensitive mode, which restricts to zero the mismatches in seed alignment, and removed the mapped reads from the sequencing files. Reads mapping the bacterial genome were highest in exponential, intermediate in dilution, and lowest in stationary stages (Fig. S1a), while reads mapping to eukaryotic rDNA operon were similar in all cases (Fig. S1b). Cleaned reads from all samples (4.9 million on average, Table S1) were co-assembled using Trinity-v2.4.0 [45]. The initial transcriptome consisted of 70,652 isoforms, for which the longest one of each gene was retained, resulting in 48,502 transcripts. These were compared using BLAST against the genome [26] and the transcriptome [25] of *C. burkhardae*, and annotated by Trinotate using UniProt [46], Pfam [47] and eggNOG [48] databases. We retained transcripts having a match to the genome or the transcriptome, or annotated as Eukaryota (19,215 left). Cleaned reads were mapped to this set with RSEM [49] and we kept 15 887 transcripts that appeared in at least 3 samples (0.3% of the signal removed). An additional BLASTn search removed obvious bacterial and viral genes (15,123 left). Transcripts with several ORFs identified by TransDecoder [45] were split when a different function was predicted for each ORF: 866 were split in two, 92 in three and 12 in four parts. The expression level of split regions was often very different (Fig. S2). Gene space completeness of the final curated transcriptome of 16,209 genes was estimated with BUSCO V3 [50].

The curated transcriptome was further processed using TRAPID [51] to annotate sequences with InterPro domains [52]. The processing strategy outlined in the original publication was slightly modified: sequence similarity search was performed using DIAMOND [53] in ‘more-sensitive’ mode (*e*-value cutoff of 10^−5^) against a stramenopile-oriented PLAZA database [54] comprising genomic data of 35 organisms including *C. burkhardae* (Table S2). Functional annotation was transferred from the top protein hit and its assigned gene family.

Cleaned reads were mapped to the curated transcriptome using RSEM. The TPM (Transcripts Per Million) table was used for sample comparison by NMDS and for DE analyses with EdgeR [55]. The latter tool detects DE genes (logFC > 2 and FDR corrected *p* values <10^−3^) in pairwise sample comparisons. InterPro domain enrichment analysis of gene sets showing a specific expression profile (e.g. genes upregulated in the exponential versus the stationary phase) was performed with TRAPID using the hypergeometric distribution, with a maximum Benjamini–Hochberg corrected *p* value cutoff of 0.05 and the entire curated transcriptome used as background. Enriched protein domains were manually assigned to given general processes and cellular functions.

## Results

### Distribution of *C. burkhardae* in the global ocean

**Fig. 1:**
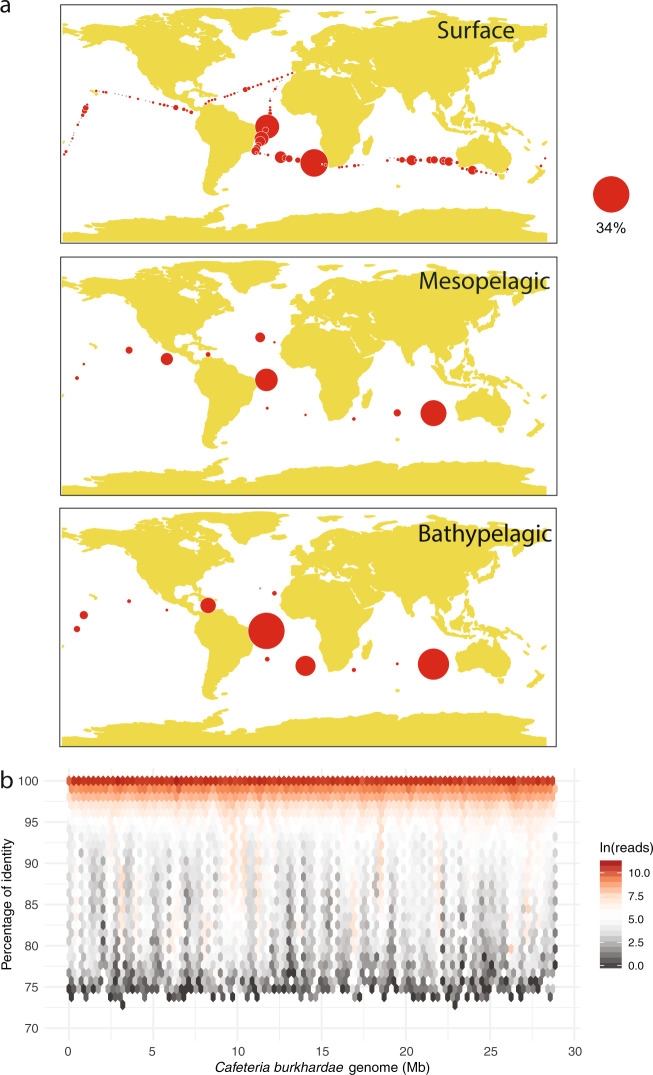
Widespread distribution of *Cafeteria burkhardae* in the global ocean. **a** Relative abundance in three vertical regions of the ASV identical to *C. burkhardae* from a study of picoplankton diversity using V4 18S rDNA amplicons. Gray circles indicate absence of the ASV, while the area of red circles is proportional to the relative abundance (the scale applies to the three panels). **b** Fragment recruitment analysis done with 66 metagenomes from the same expedition and the *C. burkhardae* genome as reference. All genome regions are mapped, with most metagenomic reads being >99% similar.

We took advantage of recently published protist diversity surveys to study the distribution of *C. burkhardae* in the global ocean (Fig. 1a). The ASV of this species was detected in most epipelagic samples (154 out of 172) with a wide variation in its relative abundance (Table 1), often below 0.1% and sometimes above 1% (median of 0.03%). The presence and relative abundance of this ASV was intermediate at the mesopelagic (found in 58 out of 61 samples; median of 0.09%) and maximal at the bathypelagic (in 58 of 60 samples; median of 0.49%). The patchy distribution of this ASV was evident in the three layers, as revealed by the huge differences between average and median values (Table 1). For instance, 22% of bathypelagic samples showed an abundance above 10%, while in 20% of samples it was below 0.1%. Performing FISH counts on 13 surface samples along the cruise track, we found cells in only 5 samples (Table 1), with abundances from 0.7 to 10.7 cells mL^−1^.

**Table 1:** Distribution of *C. burkhardae* in the global Malaspina survey by metabarcoding, metagenomics and FISH counts.

**Metabarcoding**		% of 18S rDNA genes		Distribution (% of samples)				
	Samples	Average	Median	0	<0.1	0.1–1	1–10	>10
Epipelagic (0–200 m)	172	0.74	0.03	18	47	24	9	2
Mesopelagic (200–1000 m)	61	3.41	0.09	3	49	18	20	10
Bathypelagic (1000–4000 m)	60	7.42	0.49	2	18	45	13	22

**Metagenomics**		RPM (reads per million)						
	Samples	Average	Median	Absence				
Epipelagic (0–200 m)	20	7.5	2.1	0				
Mesopelagic (200–1000 m)	26	52.8	6.2	0				
Bathypelagic (1000–4000 m)	20	801.0	30.0	0				

**FISH**		Cells mL^−1^						
	Samples	Average	Median	Absence				
Epipelagic (0–200 m)	13	1.6	0.0	8				

We then used the *C. burkhardae* genome to perform a fragment recruitment analysis against 66 metagenomes of the same expedition. This PCR-free survey detected *C. burkhardae* in all samples and confirmed the increase in relative abundance along the water column (Table 1). In three bathypelagic samples, the *C. burkhardae* genome recruited ~0.6% of reads, suggesting a high dominance of this species in their microbial assemblage that also included prokaryotes. Metagenomic reads mapped along the complete genome and were mostly placed at the 99–100% similarity interval (Fig. 1b). This occurred in the three water layers (Fig. S3), albeit at surface some genomic regions recovered reads at lower similarity, probably from highly conserved genes of other species. This metagenomic analysis indicates that the cultured strain is widespread in the global ocean.

### Dynamics of *C. burkhardae* in batch cultures

**Fig. 2:**
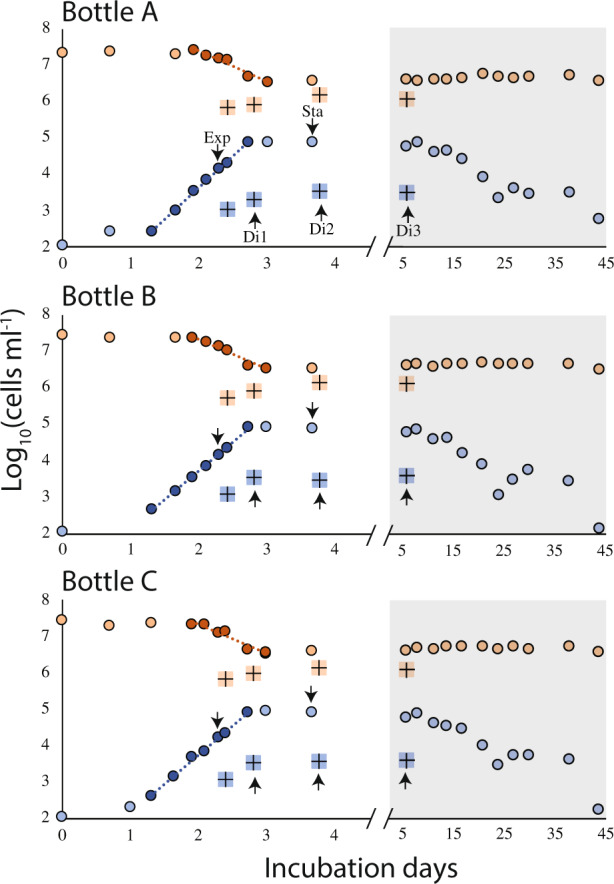
Abundance of bacteria (orange circles) and *C. burkhardae* (blue circles) in three parallel batch cultures. Points used to calculate the flagellate growth rate and the bacteria exponential decay are darker and display the derived linear regression. The abundances of both components during the dilution treatments are also shown (as colored crosses). Note the change of scale in the *x*-axis at the shaded area. Samples for transcriptomics are marked with an arrow.

**Fig. 3:**
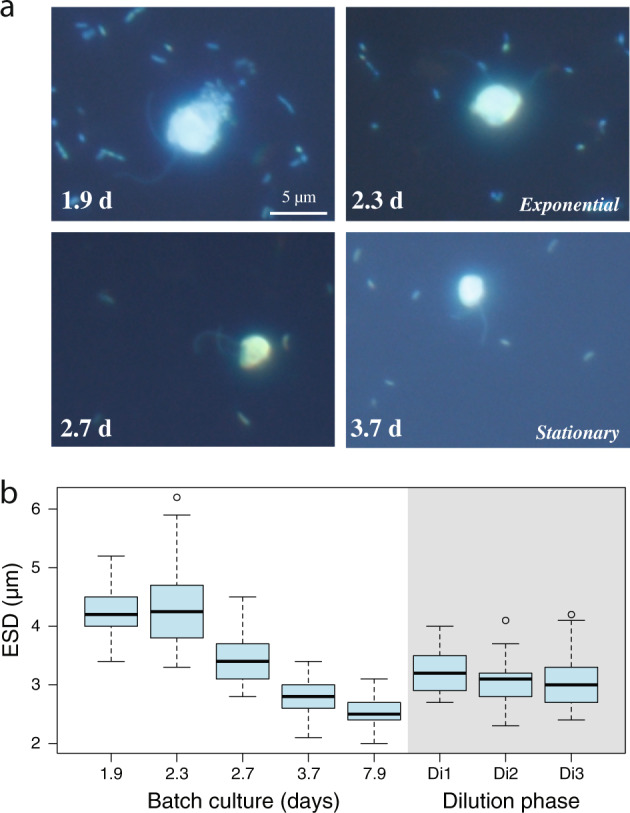
Cell size changes of *C. burkhardae* at different growth states. **a** Epifluorescence microscope images of flagellates and bacteria in different days of the batch culture. The scale bar applies to all images. **b** Box plots of the ESD (Equivalent Spherical Diameter) of about 50 cells during the batch culture and in the three dilution events.

The cell dynamics of *C. burkhardae* and *Dokdonia* MED134 in the three batch cultures were highly reproducible (Fig. 2). After a short latency phase, there was a very fast growth of the flagellate population, so that over a 34 h period densities increased from a few hundreds to 8 × 10^4^ cells mL^−1^ in a perfect exponential growth curve (*R*^2^ ≥ 0.99), yielding doubling times of 4.2–4.6 h (Table S3). Parallel to the flagellate growth there was an exponential decay of bacteria, whose abundance fell from 25 to 3.5 × 10^6^ cells mL^−1^. The grazing rates in the three cultures were 40–49 bacteria flagellate^−1^ h^−1^, and the estimated growth efficiencies were ~40%. Cultures remained relatively stable after the exponential phase, with similar bacterial numbers for weeks and a slow decrease of flagellate numbers, with half-life exponential decay of 121–140 h. Flagellate cell size changed during the batch culture (Fig. 3), with larger cells at the exponential phase than at the stationary phase.

The three batch cultures were diluted 20-fold in the middle of the exponential phase to reduce bacterial abundances below the level supporting flagellate growth. Cell counts at different times after the dilution showed one or two divisions of the flagellate population, likely at the expense of what they had ingested before dilution, until they stopped growing (Fig. 2). Bacterial counts doubled only once, indicating no bacterial growth in sterile seawater. Flagellate cell sizes at the different dilution times were in between the exponential and stationary states (Fig. 3b). We regarded these dilutions as a different way of entering starvation, more gradual than the abrupt stationary state.

### De novo transcriptome of *C. burkhardae* and overall expression profiles

Gene expression analysis was performed in 21 samples from six phases: the exponential phase, the stationary phase, three states after starving by dilution for different times, and the inoculum (Table S1). Each phase included a mix of biological replicates (different bottles) and technical replicates (same bottle). Poor quality raw Illumina reads and those mapping the *Dokdonia* sp. genome or the *C. burkhardae* rDNA operon were removed, leaving only about one third of the reads. These were assembled to generate a de novo transcriptome, which was then curated to keep transcripts with a high likelihood to belong to *C. burkhardae* based on genomic data, transcriptomic data, and functional annotations. The de novo transcriptome had 16,209 genes and an estimated BUSCO completeness of 82.2% (for comparison, the annotated genome has a BUSCO score of 83.8%, [26]).

**Fig. 4:**
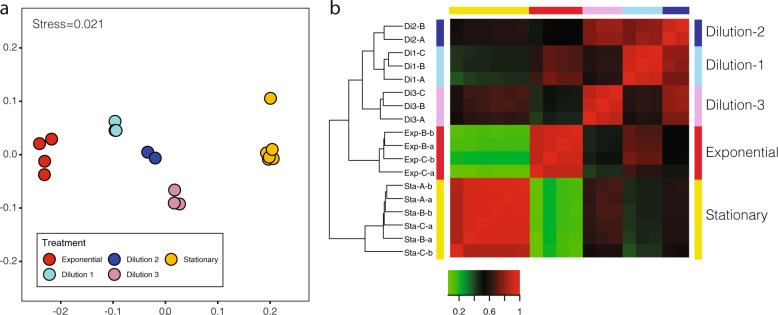
Comparison of the expression profiles of all samples in the five main states. **a** NMDS (nonmetric multidimensional scaling) plot placing samples in a two dimensional space based on TPM values of all genes. **b** Heatmap showing Pearson correlation coefficients in sample pairwise comparisons based on differently expressed genes.

Cleaned reads were mapped to the de novo transcriptome to get the TPM values of each transcript per sample (74.3% mapped reads on average, Table S1). We focused on the expression profiles of the five phases derived from well-controlled conditions. Samples from the same phase grouped together, while each phase occupied a different position in the NMDS plot (Fig. 4a). The three dilution events were placed orderly between exponential and stationary phases, following an apparent temporal trend of transcriptional activity. We then computed the differentially expressed (DE) genes between all phases (Table S4). Grouping of samples based on DE genes was consistent with their NMDS placement and showed that biological and technical replicates were indistinguishable, with Pearson correlation coefficients close to 1 (Fig. 4b), so they could all be treated as replicates of the experimental condition. Further analyses including the Inoculum and the MMETSP transcriptome (for which the culture state was undetermined) showed these two states were far from exponential samples (Fig. S4). In particular, the Inoculum was placed between dilution-3 and stationary, while the MMETSP had a more distant position.

### Differentially expressed genes and highly expressed genes

As the exponential to stationary pair presented the highest number of DE genes, with 1231 and 825 upregulated genes, respectively, an enrichment analysis was performed to identify the biological functions associated to these DE gene sets (Table 2). Enriched functions among genes upregulated during the exponential phase invoked a population of actively dividing cells, with proteins involved in DNA replication (structural maintenance of chromosome), transcription and RNA processing (RNA helicases, exoribonucleases), and protein remodeling (heat shock proteins). Phagocytosis was the other general process enriched in the exponential phase, represented by digestive enzymes (peptidases M16 and S53), and proton pumps (V-PPase). Among genes upregulated during the stationary phase there was a striking enrichment of functions related to signaling and cell response, in particular signal transduction (histidine kinases) and cell adhesion (VWF and extracellular protein domains like EGF, laminin or lectin). Other intriguing functions enriched in the stationary phase were those related to lipid metabolism (fatty acid desaturases).

**Table 2:** Enriched functions based on InterPro domains in the subset of upregulated genes at the exponential phase (1231) or the stationary phase (825) as compared with the complete transcriptome.

**Enriched functions among genes upregulated during the exponential phase**						
InterPro entry	Enrichment fold	Adjusted *p* value	Subset ratio	Description	General process	Cellular function
IPR024704	2.75	0.021	0.50	Structural maintenance of chromosomes protein	Information processing	DNA replication and repair
IPR003395	2.73	0.006	0.79	RecF/RecN/SMC, N-terminal	Information processing	DNA replication and repair
IPR031327	2.54	0.006	0.69	Mini-chromosome maintenance protein	Information processing	DNA replication and repair
IPR018314	3.43	0.046	0.30	Eukaryotic nucleolar NOL1/Nop2p	Information processing	Transcription and RNA processing
IPR001247	3.43	0.046	0.30	Exoribonuclease, phosphorolytic domain 1	Information processing	Transcription and RNA processing
IPR014014	1.55	0.008	1.49	RNA helicase, DEAD-box, Q motif	Information processing	Transcription and RNA processing
IPR000629	1.40	0.047	1.29	ATP-dependent RNA helicase DEAD-box	Information processing	Transcription and RNA processing
IPR011545	1.11	0.008	2.68	DEAD/DEAH box helicase	Information processing	Transcription and RNA processing
IPR014001	0.93	0.005	4.06	Helicase superfamily 1/2	Information processing	Transcription and RNA processing
IPR015366	2.58	0.040	0.50	Peptidase S53	Phagocytosis	Digestive enzyme
IPR001431	2.55	0.015	0.59	Peptidase M16, zinc-binding site	Phagocytosis	Digestive enzyme
IPR007863	2.28	0.005	0.89	Peptidase M16	Phagocytosis	Digestive enzyme
IPR004131	3.43	0.046	0.30	V-PPase	Phagocytosis	Proton pump
IPR019805	3.43	0.003	0.50	Heat shock protein 90	Protein cellular processes	Protein folding
IPR018181	2.62	0.002	0.79	Heat shock protein 70	Protein cellular processes	Protein folding
**Enriched functions among genes upregulated during the stationary phase**						
InterPro entry	Enrichment fold	Adjusted *p* value	Subset ratio	Description	General process	Cellular function
IPR003349	4.43	0.044	0.40	JmjN domain	Information processing	Transcription and RNA processing
IPR011388	3.69	0.025	0.60	Sphingolipid delta4-desaturase	Metabolism	Lipid metabolism
IPR005804	2.62	0.009	1.19	Fatty acid desaturase	Metabolism	Lipid metabolism
IPR000323	4.43	0.044	0.40	Ascorbate-dependent monooxygenase	Metabolism	Oxidoreductase activity
IPR006595	4.43	0.004	0.60	C-terminal LisH motif	Motility and cytoskeleton	Cytoeskeleton
IPR000203	3.69	0.025	0.60	GPS motif	Protein cellular processes	Protein modification
IPR023313	2.58	0.025	0.99	Ubiquitin-conjugating enzyme	Protein cellular processes	Protein modification
IPR001791	4.43	0.004	0.60	Laminin G domain	Signaling and cell response	Cell adhesion
IPR001220	4.43	0.001	0.79	Legume lectin domain	Signaling and cell response	Cell adhesion
IPR001846	4.43	0.004	0.60	von Willebrand factor, type D domain	Signaling and cell response	Cell adhesion
IPR026588	4.43	0.004	0.60	Choice-of-anchor A domain	Signaling and cell response	Cell adhesion
IPR019316	4.43	0.004	0.60	G8 domain	Signaling and cell response	Cell adhesion
IPR002909	1.62	0.002	3.57	IPT domain	Signaling and cell response	Cell adhesion
IPR000742	1.44	0.018	2.78	EGF-like domain	Signaling and cell response	Cell adhesion
IPR029927	4.43	0.004	0.60	Fibrocystin-L	Signaling and cell response	Signal transduction
IPR003661	2.17	<0.001	4.96	Histidine kinase, dimerization	Signaling and cell response	Signal transduction
IPR005467	2.13	0.001	2.58	Histidine kinase domain	Signaling and cell response	Signal transduction
IPR004358	1.95	0.004	2.38	Histidine kinase, C-terminal	Signaling and cell response	Signal transduction
IPR003594	1.78	<0.001	4.56	Histidine kinase/HSP90-like ATPase	Signaling and cell response	Signal transduction
IPR001789	1.94	<0.001	4.96	Signal transduction response regulator	Signaling and cell response	Signal transduction
IPR002110	0.75	0.026	7.54	Ankyrin repeat	Signaling and cell response	Signal transduction

Enrichment fold values are reported in log_2_ scale. The subset ratio indicates the percentage of DE genes within each function.

**Fig. 5:**
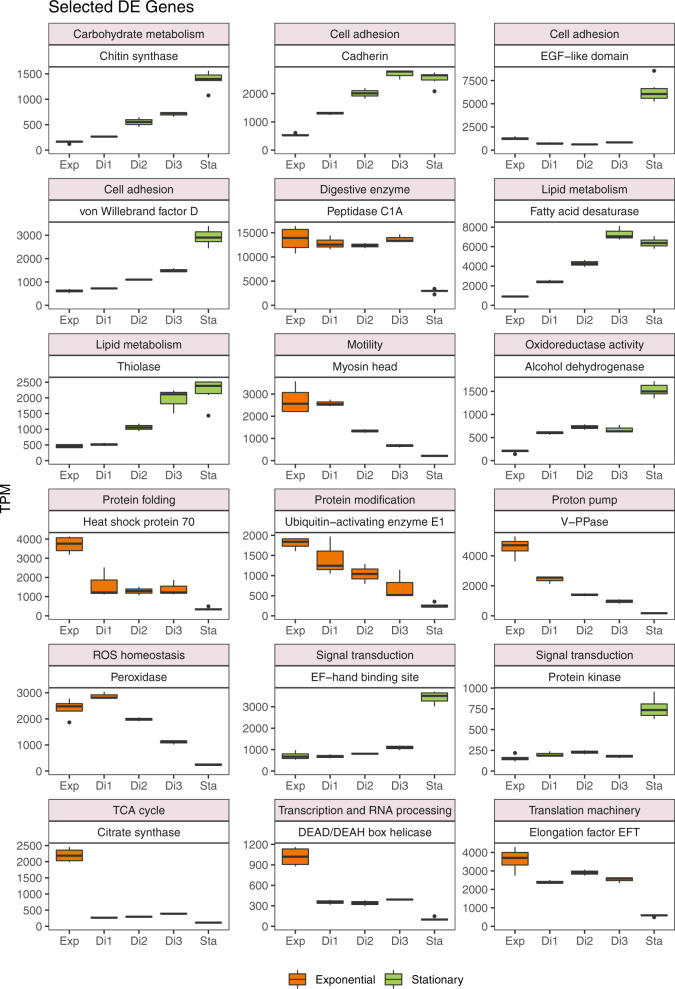
Box plots displaying the transcriptional changes along the five states of a few highly expressed genes. Genes are selected because they are differentially expressed in exponential versus stationary phases and appeal for important cellular functions.

We finally focused on the most highly expressed genes, those with an average TPM value >500 in any of the five phases. The selected 432 genes accounted for a considerable share of the expression signal in all samples (from 52 to 66%; 62% on average) and were manually assigned to a cellular function included in a general process. Comparing the exponential and stationary phases, we found that 79 of these highly expressed genes were upregulated in the exponential phase, 94 in the stationary phase, and 259 were similarly expressed. These genes generally followed a regular expression pattern from exponential to stationary, with the dilution phases in between (Fig. S5). From this list, we selected a few relevant genes that may be optimal cornerstones to study specific process (Fig. 5). The function of many of them corresponded to the enriched functions found before (Table 2), and we also point to additional cases of genes upregulated in the exponential phase (myosin, ubiquitin, elongation factor, peroxidase), or in the stationary phase (chitin synthase, thiolase, cadherin, dehydrogenase).

**Fig. 6:**
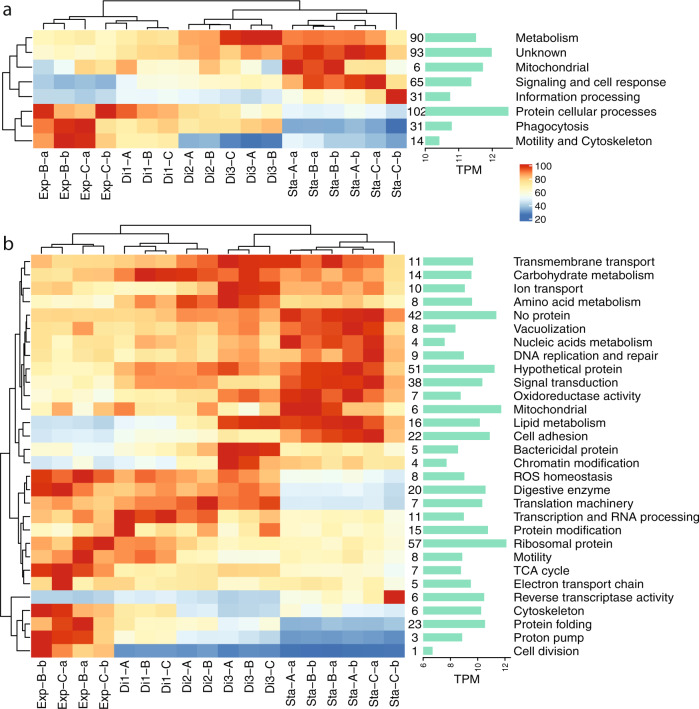
Overall gene expression changes at a broad functional level. Gene expression changes of general processes (**a**) and associated cellular functions (**b**) computed by adding up the TPM values of highly expressed genes within these categories (numbers of genes per category shown after the heatmap). The data displayed in each cell represent the percentage with respect to the highest value in the process/function (considered 100%). Bar plots on the right display the actual TPM value of this highest cell.

The classification of highly expressed genes in functional categories allowed us to analyze functional expression changes in the different states (by adding up the TPM values of genes within each category). On a broad level (Fig. 6a), there were several general processes that decreased their expression from exponential, through dilutions to the stationary phase: protein cellular processes (which displayed the highest expression), phagocytosis, motility, and cytoskeleton. The remaining general processes exhibited the opposite trend. On a more specific level (Fig. 6b), cellular functions that reduced their expression from exponential to stationary formed two groups, those with a sudden decrease (cytoskeleton, protein folding, and proton pump) and those with a gradual decrease (transcription and translation machinery, TCA cycle, digestive enzymes, motility). Genes stimulated during starvation also displayed two distinct groups: those with a highly increased expression (lipid metabolism, cell adhesion, bactericidal proteins) and those with a moderate increase (transporters, amino acid and carbohydrate metabolism, signal transduction).

## Discussion

### An opportunistic and widely distributed heterotrophic flagellate

Marine microbial ecology has accepted the “uncultured majority” problem [56], where many ecologically relevant species are uncultured, and as a result we lack optimal ecophysiological models to interpret ecosystem processes. The genus *Cafeteria* was described decades ago [23], is easily cultured from marine samples [5], but was considered to be of little ecological relevance [29]. The analysis of sequencing data from the global Malaspina expedition, however, showed that *C. burkhardae* was a widespread species, often at very low abundance but with a few cases of high abundance. This patchiness contrasted with the log-normal distribution of other uncultured heterotrophic flagellates [35]. Its relative abundance increased through the water column, which does not need to imply an increase in cell counts, because of the drastic decrease of heterotrophic flagellates numbers with depth [57]. In addition, the metagenomic signal in the open sea matched perfectly with the genome of the cultured strain, indicating that this strain is a good representative of a widespread marine species.

Batch cultures allow a simple and quick evaluation of the growth and grazing kinetics of heterotrophic flagellates. In our cultures, *C. burkhardae* was a fast growing and ferocious predator, with grazing (50 bacteria h^−1^) and growth rates (0.16 h^−1^) comparable to the rates of cultured heterotrophic flagellates [27, 58]. Grazing rates of cultured species are higher than typical community rates, 2–20 bacteria h^−1^ [3]. *C. burkhardae* had a long survival at the stationary phase, with thousands of cells mL^−1^ still present after 40 days. Another interesting aspect was that the growth ceased at bacterial abundances of 3 × 10^6^ cells mL^−1^, a density higher than typical bacterioplankton abundances of 10^5^–10^6^ cells mL^−1^ in surface and 10^4^–10^5^ in deep waters. This suggests that *C. burkhardae* may grow in patches of high food abundance, such as those found in permanent or ephemeral particles [59, 60]. The increase in cell volume during fast growth can be an adaptation to exploit temporary enriched environments. After explosive growth, this species can survive for weeks until a new particle is colonized. This feast and famine existence [61] is consistent with its patchy distribution and its increase with depth, as the relative importance of particles in microbial processes seems to increase with depth [62].

### Transcriptional profiles in different physiological states

Transcriptomics is a promising and accessible way to gather new evolutionary and ecological insights into microbial eukaryotes [10], but few studies have been done with bacterivorous flagellates [21, 63, 64]. In some cases, the transcriptome is designed to retrieve genes for multigene phylogenies and, as seen here, many genes are expressed in all growth states. To fulfill our aim of identifying genes involved in phagocytosis, it was essential to link gene expression with the growth status. Accordingly, we put a considerable effort into sampling the exponential phase, which was challenging because only few hours separated the start of apparent growth and the stationary phase. Without a dedicated microscopic inspection, it would have been easy to miss this short window of time and sample dense and stationary cultures. That was likely the case for the MMETSP sample (and most bulk transcriptomes focused on gene discovery) that had a transcriptional profile closer to stationary samples. We also artificially “synchronized” cells to a gradual transition to starvation by dilution (by reducing bacterial encounter). The dilution samples had distinct expression profiles and were placed in an ordered manner between exponential and stationary phases (Figs. 4–6).

We identified a large number of genes (12.7% of total) that were differentially expressed between the exponential and stationary phases. Many of the DE genes upregulated in the exponential phase were related to the functions expected in the scenario of a population of cells feeding, converting food to biomass and dividing: DNA replication, transcription, translation, protein modification, respiration, cytoskeleton reorganization, and phagocytosis. In the stationary state, when cells had miniaturized to adapt to starvation, many upregulated genes related to signaling and cell response, with signal transduction across membranes and cell adhesion being the most significant, suggesting a crucial role in sensing the environment for hotspots to restart grazing and growth. The gene coding for fatty acid desaturase, which forms double bonds in fatty acids to increase membrane fluidity [65], was upregulated in the stationary phase, perhaps to accommodate extracellular protein domains like cadherin, lectin, and laminin in the membrane, also upregulated at this phase. Also intriguing was the high expression of chitin synthase, a gene that has been found in other stramenopiles that were not thought to contain chitin [66]. It could be speculated that chitin might provide cell rigidity to this species, contributing to its survival during starvation. Finally, many unknown genes were highly expressed (Fig. S5), some with homologous in other eukaryotes (hypothetical protein; 51 genes) and others with no match at all (no protein; 42 genes). More than half were differentially expressed, some upregulated at the exponential (11 genes) but the majority at the stationary (57 genes). These unknown DE genes represent interesting grounds for future functional genomics explorations.

### Upregulated genes in exponential state targeting phagocytosis

Phagocytosis is a very complex process involving the coordinated action of many proteins [16]. It is of great evolutionary and ecological significance, so one major aim of our study was to identify highly expressed genes functionally related to phagocytosis. The upregulated gene in the exponential phase with the highest expression level coded for a digestive enzyme of the Peptidase C1A family, a group of cysteine peptidases that typically include lysosomal or secreted proteins [67, 68]. The majority of cathepsins, known to be activated in the acidic lysosomes, belong to this family. Other peptidases were also highly expressed in the exponential phase: Peptidase S53, a serine peptidase with optimal pH of 3, and Peptidase M16, a metal dependent peptidase. Other upregulated digestive enzymes were adenosylhomocysteine hydrolase, which hydrolyzes the biosynthetic precursor of homocysteine, and the alpha/beta hydrolase fold that is common to hydrolytic enzymes of varied catalytic function.

Digestive enzymes used in phagocytosis operate in the acidic environment of mature phagosomes, which are acidified by the action of the transmembrane proton pumps V-ATPases and V-PPases [69]. Although both types were found in *C. burkhardae*, the V-PPase (vacuolar pyrophosphatase) exhibited a higher expression, being the fifth most highly expressed gene in the exponential state. So, this proton pump seems to be responsible for phagosome acidification in this species. In a recent experiment we identified a high expression of rhodopsin in the uncultured MAST-4 heterotrophic flagellate [70], and hypothesized that the coding protein acted as a light-driven proton pump that contributed to phagosome acidification. Even though rhodopsin genes were found in the *C. burkhardae* transcriptome, they were never highly expressed. This may explain why this species is not restricted to photic waters.

Finally, two of the highly expressed genes in the exponential phase were peroxidases. The canonical function of these enzymes is to detoxify deleterious reactive oxygen species (ROS). In the reverse action, peroxidases can produce ROS radicals, which in phagocytes of the animal immune system participate in killing pathogens [71]. In free-living protists that use phagocytosis for nutrition, such as the amoebozoan *Dictyostelium*, the involvement of ROS radicals in prey processing has not been demonstrated [18], but our data suggest they may possibly play a role in prey digestion, although this is currently speculative.

## Concluding remarks

Functional and genomic analyses with marine bacterivorous heterotrophic flagellates have been limited by the lack of appropriate model species. Using molecular diversity surveys, we show that the well-known cultured species *C. burkhardae* is widespread in the ocean and seems to be an opportunistic species that grows fast in patches of high bacterial density and becomes a good survivor in the diluted surrounding seawater. In batch cultures, *C. burkhardae* presents marked changes in gene expression when actively growing and when starving, and we identified promising gene sets specific for each state. Whether or not these match with the genetic machinery at play in natural communities, where this species faces complex biotic and abiotic interactions, remains an open question. Among the most interesting genes during active grazing are those related to phagocytosis, such as digestive enzymes, proton pumps, and perhaps peroxidases. Future studies with other cultured heterotrophic flagellates, or even more interestingly with natural or manipulated assemblages [70], will be necessary to evaluate if these genes are functionally relevant in other species as well, in which case they will represent promising markers to study bacterivory in the oceans.

## Supplementary information

Supplementary Material
