# Supplementary material for: Gene expression during bacterivorous growth of a widespread marine heterotrophic flagellate
Source: ISME J. 2020 Sep 12;15(1):154–67. doi: 10.1038/s41396-020-00770-4 (PMC7852580; doi:10.1038/s41396-020-00770-4)
Supplement: Supplementary file 1 — Supplementary Material [file 41396_2020_770_MOESM1_ESM.pdf]

Supplementary Information

Fig. S1 Cleaning of sequencing reads before *de novo* transcriptome assembly. **a** Percentage of reads mapping the *Dokdonia* MED134 genome. **b** Percentage of reads mapping the operon of *C. burkhardae* (after excluding *Dokdonia* reads).

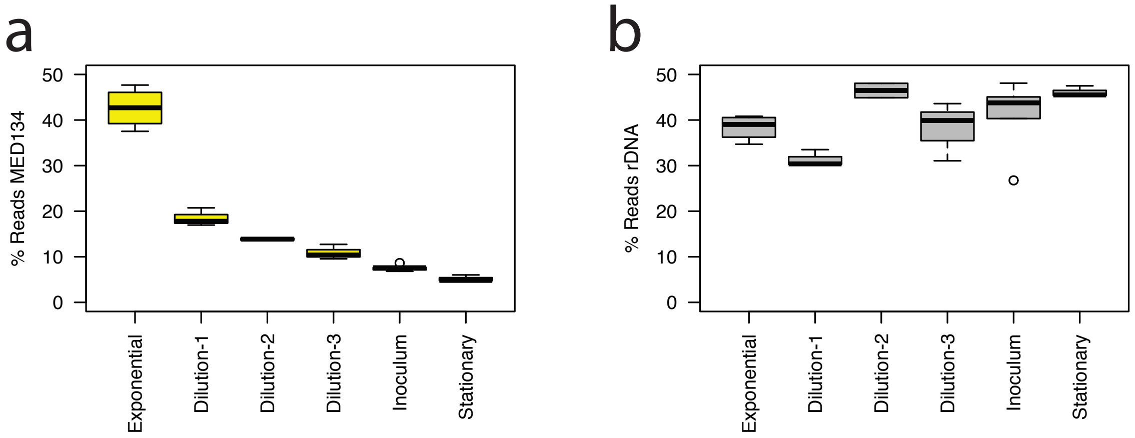

Fig. S2 Expression level of selected transcripts before and after being split in 2-4 fragments based on the presence of ORFs with different functional predictions. The list shows highly expressed transcripts in the Exponential phase (average TPM >500 before splitting).

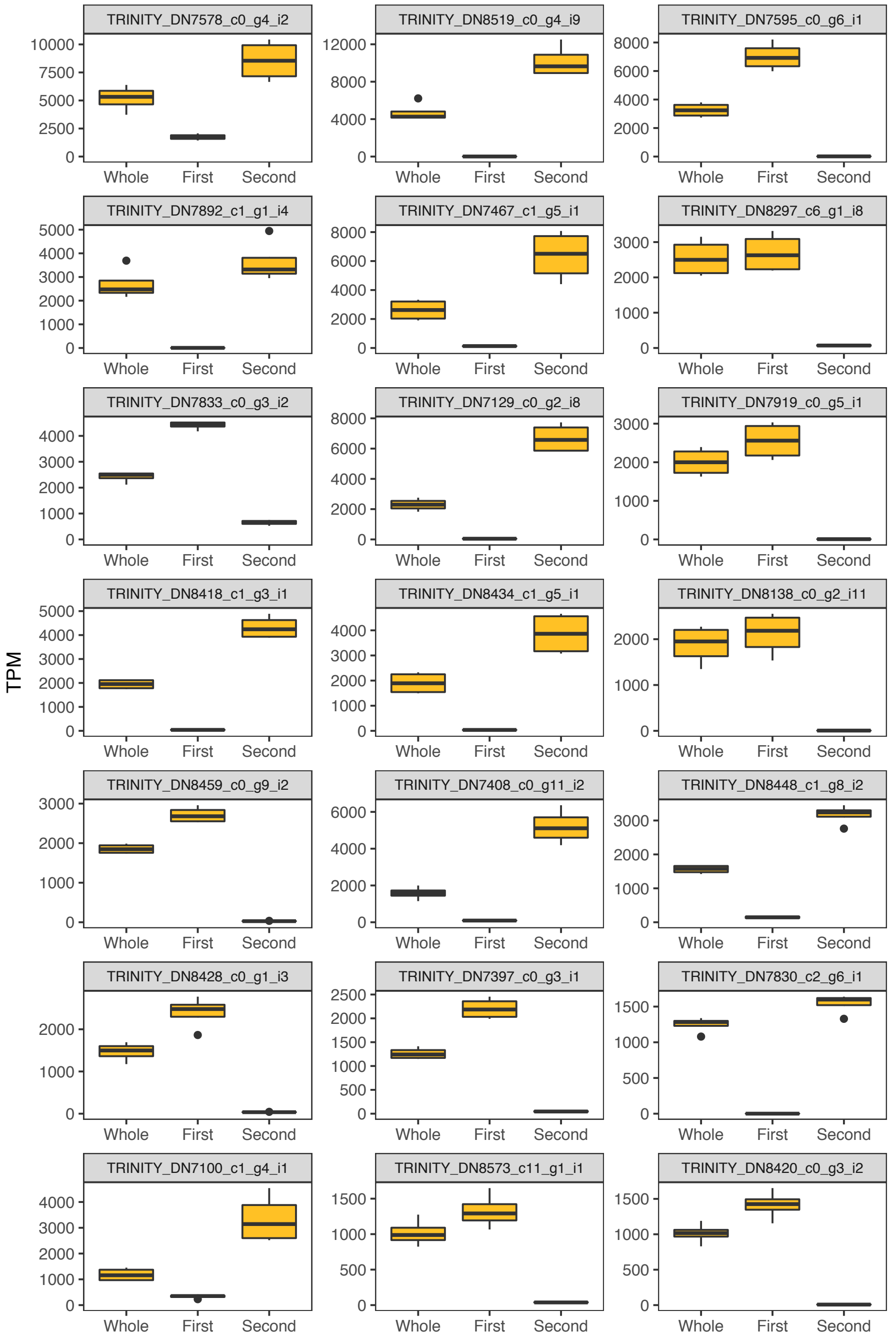

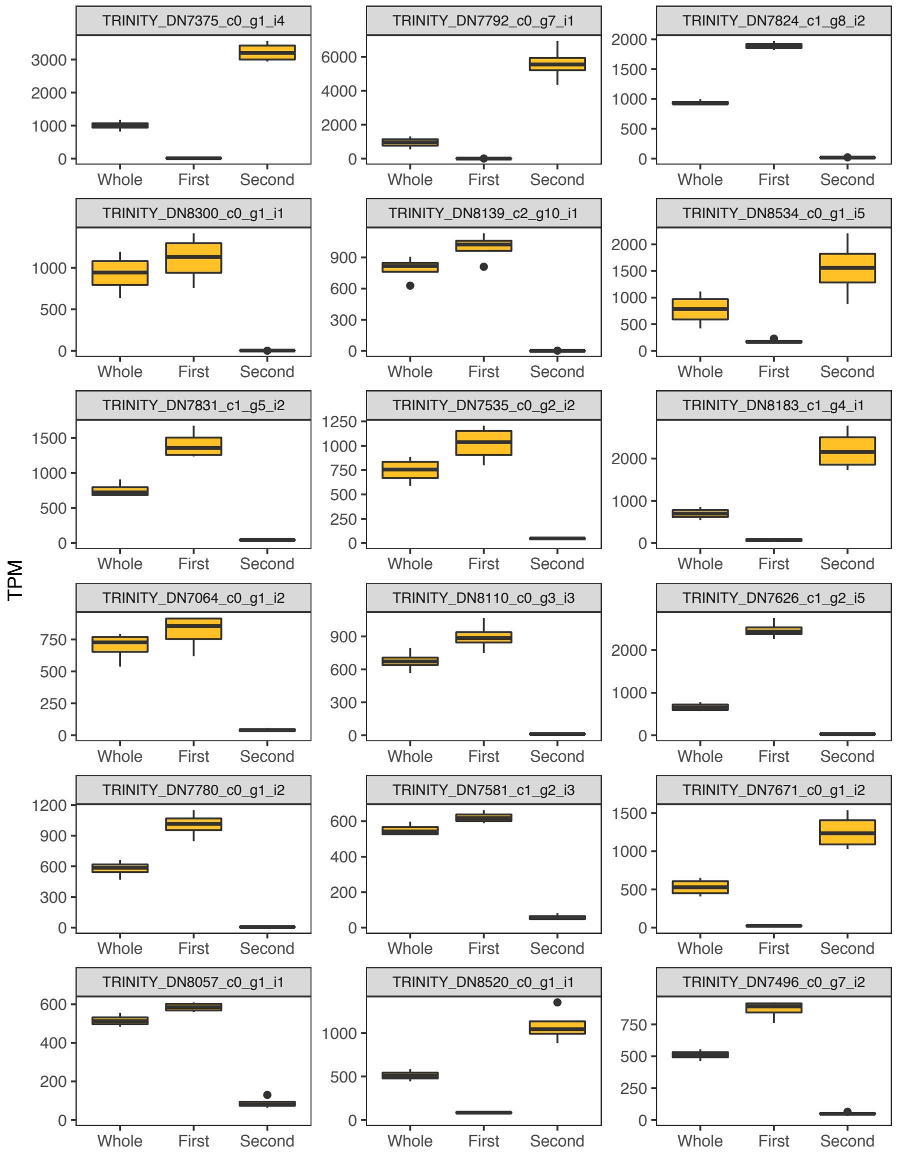
TRINITY_DN7375_c0_g1_i4
TRINITY_DN7792_c0_g7_i1
TRINITY_DN7824_c1_g8_i2
TRINITY_DN8300_c0_g1_i1
TRINITY_DN8139_c2_g10_i1
TRINITY_DN8534_c0_g1_i5
TRINITY_DN7831_c1_g5_i2
TRINITY_DN7535_c0_g2_i2
TRINITY_DN8183_c1_g4_i1
TRINITY_DN7064_c0_g1_i2
TRINITY_DN8110_c0_g3_i3
TRINITY_DN7626_c1_g2_i5
TRINITY_DN7780_c0_g1_i2
TRINITY_DN7581_c1_g2_i3
TRINITY_DN7671_c0_g1_i2
TRINITY_DN8057_c0_g1_i1
TRINITY_DN8520_c0_g1_i1
TRINITY_DN7496_c0_g7_i2
TPM
Whole
First
Second

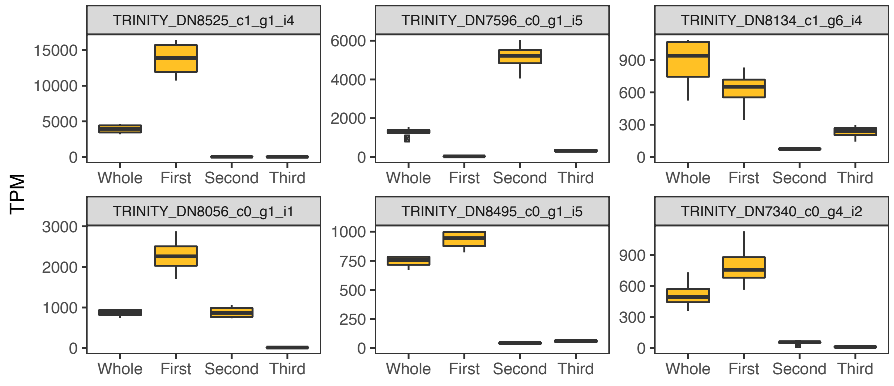
TRINITY_DN8525_c1_g1_i4
TRINITY_DN7596_c0_g1_i5
TRINITY_DN8134_c1_g6_i4
TRINITY_DN8056_c0_g1_i1
TRINITY_DN8495_c0_g1_i5
TRINITY_DN7340_c0_g4_i2
TPM
Whole
First
Second
Third

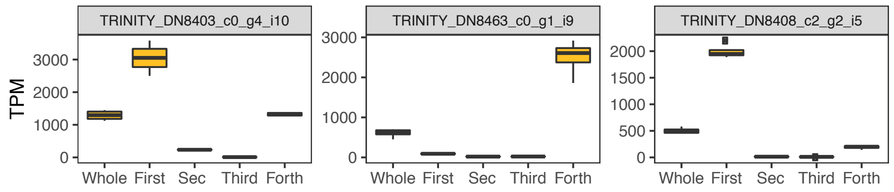
TRINITY_DN8403_c0_g4_i10
TRINITY_DN8463_c0_g1_i9
TRINITY_DN8408_c2_g2_i5
TPM
Whole
First
Sec
Third
Forth

Fig. S3 Fragment recruitment analysis done with metagenomes from the Malaspina expedition and the *C. burkhardae* genome as reference. Data is separated in the three water column regions, epipelagic (20 metagenomes), mesopelagic (26), and bathypelagic (20).

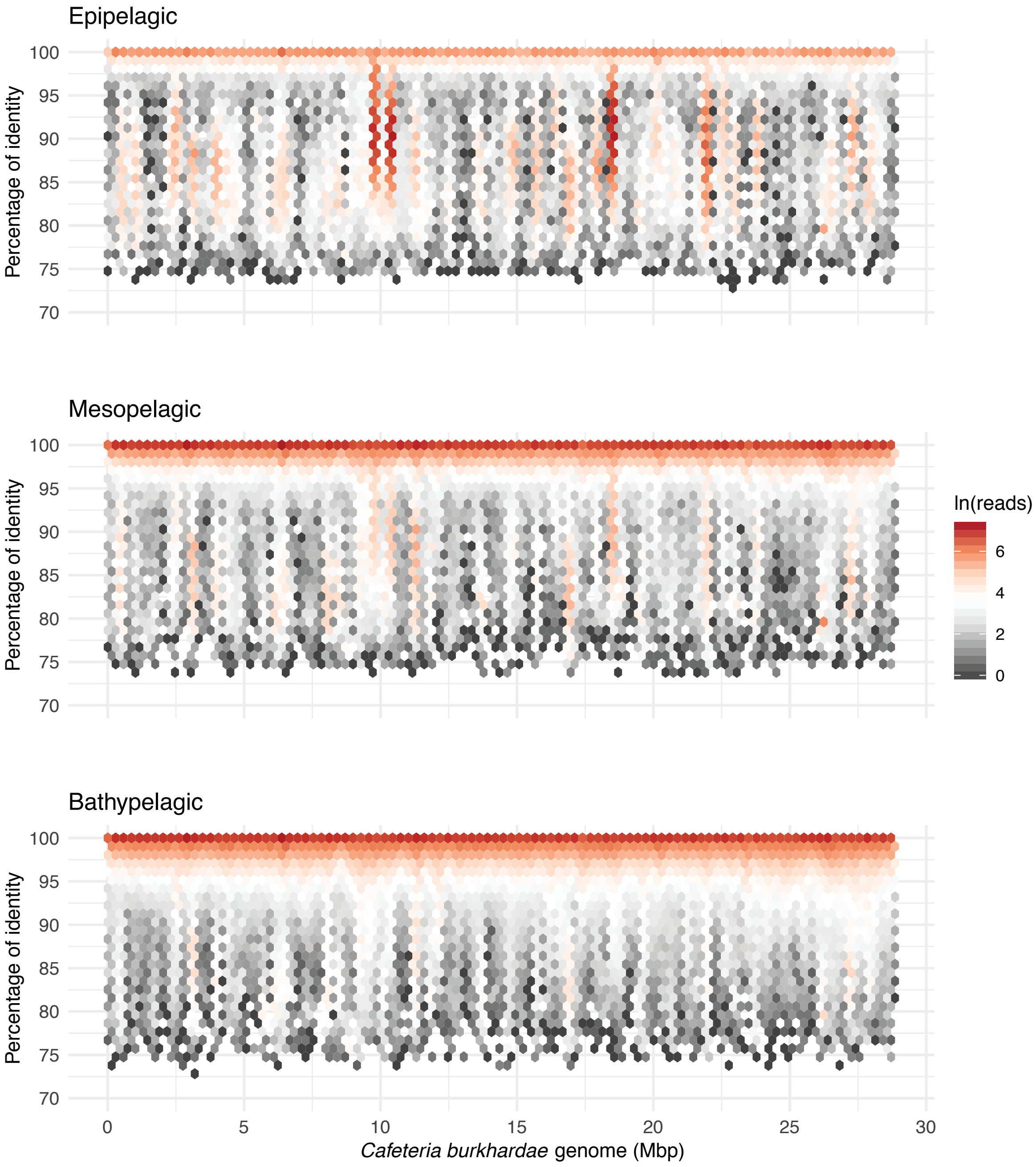

Fig. S4 Comparison of the expression profiles of all samples in the five main states plus the Inoculum, and the MMETSP transcriptome. **a** NMDS plot placing samples in a two dimensional space based on TPM values of all genes. **b** Heatmap showing Pearson correlation coefficients in sample pairwise comparisons based on differently expressed genes.

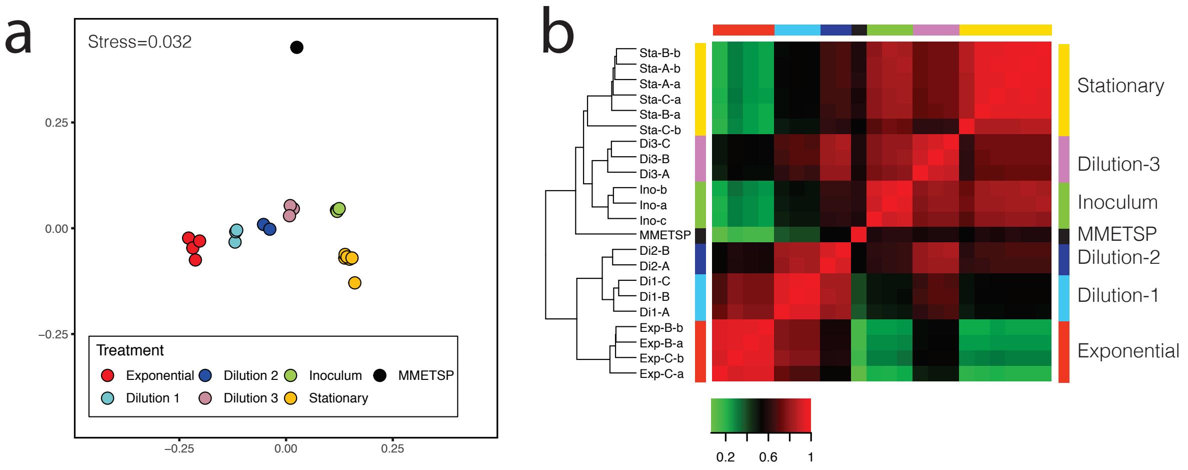

Fig. S5 Box plots displaying expression changes in the five states of the 432 highly expressed genes, ordered based on their general process and cellular function and then by differential expression between Exponential and Stationary. Orange: genes upregulated in Exponential; Green; genes upregulated in Stationary; Grey: genes equally expressed.

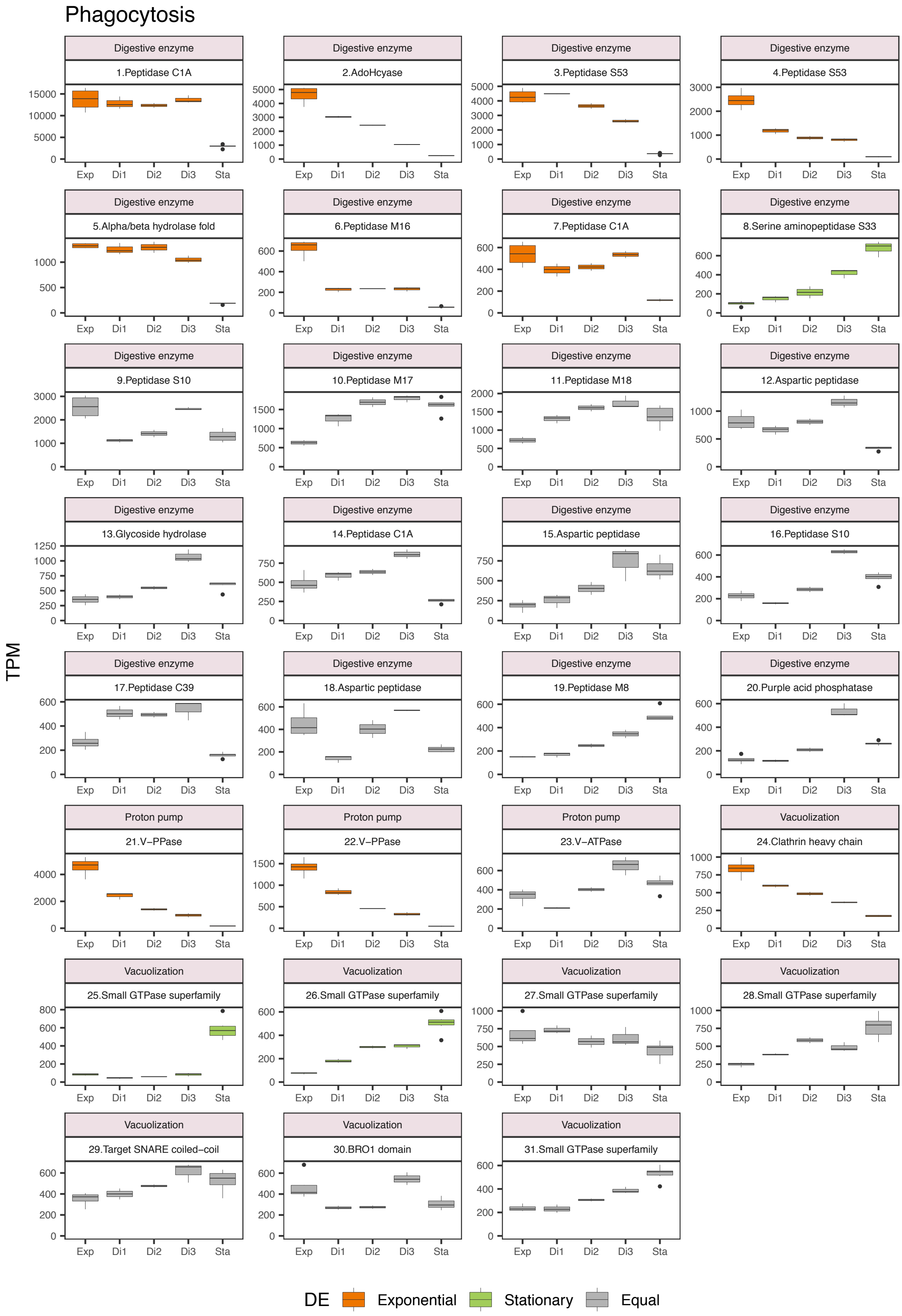

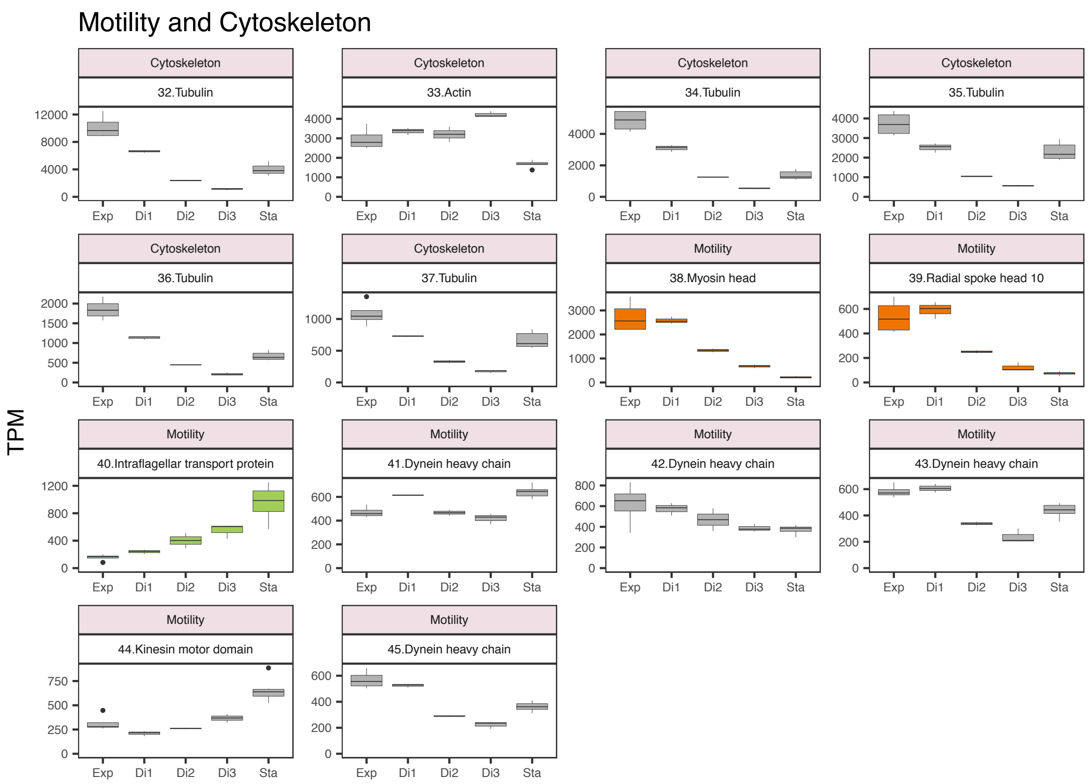
Motility and Cytoskeleton
TPM
Cytoskeleton
32.Tubulin
Cytoskeleton
33.Actin
Cytoskeleton
34.Tubulin
Cytoskeleton
35.Tubulin
Cytoskeleton
36.Tubulin
Cytoskeleton
37.Tubulin
Motility
38.Myosin head
Motility
39.Radial spoke head 10
Motility
40.Intraflagellar transport protein
Motility
41.Dynein heavy chain
Motility
42.Dynein heavy chain
Motility
43.Dynein heavy chain
Motility
44.Kinesin motor domain
Motility
45.Dynein heavy chain
Exp
Di1
Di2
Di3
Sta

Information processing

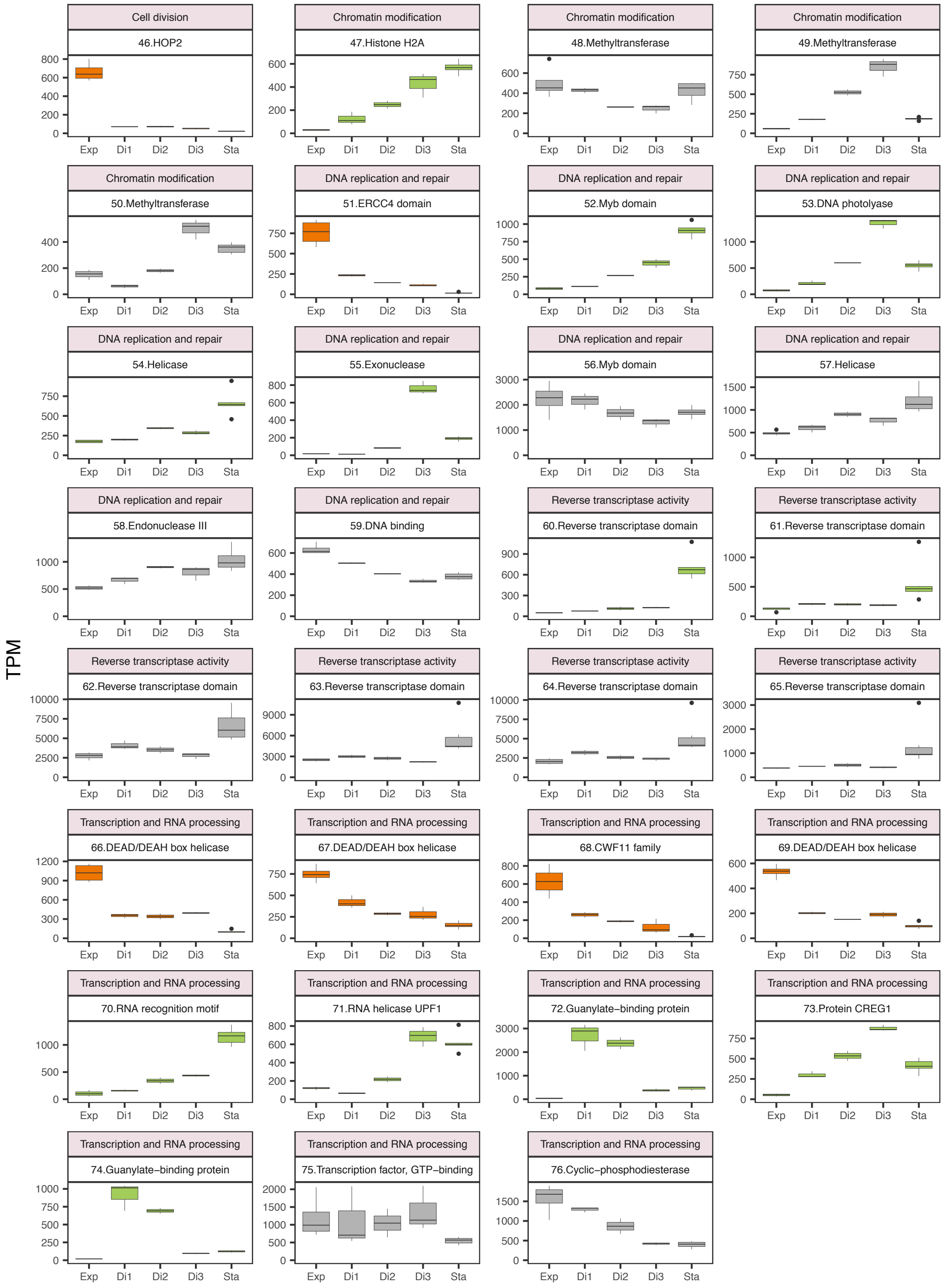

Metabolism

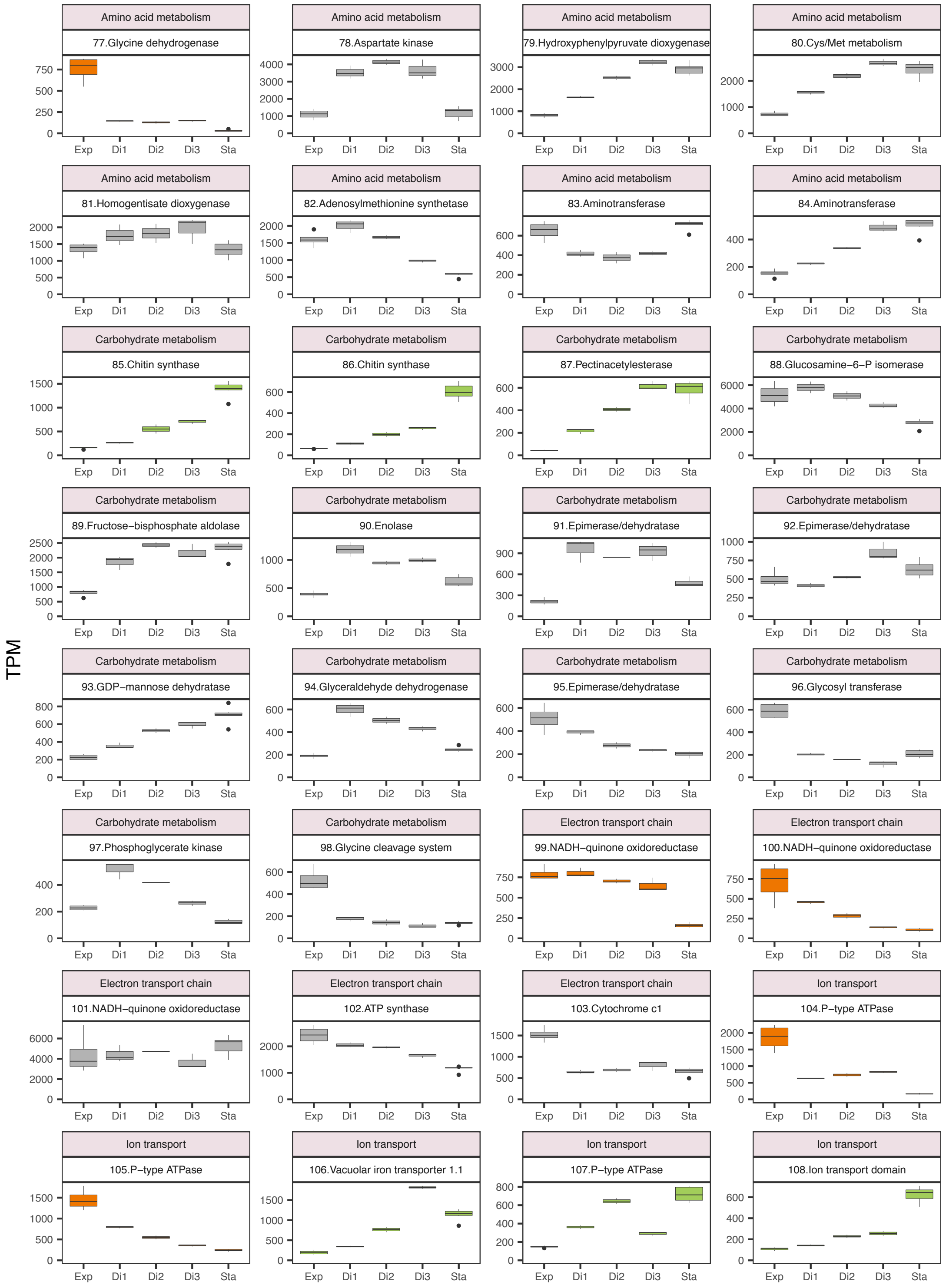
TPM
Exp Di1 Di2 Di3 Sta
Amino acid metabolism
77.Glycine dehydrogenase
Amino acid metabolism
78.Aspartate kinase
Amino acid metabolism
79.Hydroxyphenylpyruvate dioxygenase
Amino acid metabolism
80.Cys/Met metabolism
Amino acid metabolism
81.Homogentisate dioxygenase
Amino acid metabolism
82.Adenosylmethionine synthetase
Amino acid metabolism
83.Aminotransferase
Amino acid metabolism
84.Aminotransferase
Carbohydrate metabolism
85.Chitin synthase
Carbohydrate metabolism
86.Chitin synthase
Carbohydrate metabolism
87.Pectinacetylesterase
Carbohydrate metabolism
88.Glucosamine−6−P isomerase
Carbohydrate metabolism
89.Fructose−bisphosphate aldolase
Carbohydrate metabolism
90.Enolase
Carbohydrate metabolism
91.Epimerase/dehydratase
Carbohydrate metabolism
92.Epimerase/dehydratase
Carbohydrate metabolism
93.GDP−mannose dehydratase
Carbohydrate metabolism
94.Glyceraldehyde dehydrogenase
Carbohydrate metabolism
95.Epimerase/dehydratase
Carbohydrate metabolism
96.Glycosyl transferase
Carbohydrate metabolism
97.Phosphoglycerate kinase
Carbohydrate metabolism
98.Glycine cleavage system
Electron transport chain
99.NADH−quinone oxidoreductase
Electron transport chain
100.NADH−quinone oxidoreductase
Electron transport chain
101.NADH−quinone oxidoreductase
Electron transport chain
102.ATP synthase
Electron transport chain
103.Cytochrome c1
Ion transport
104.P−type ATPase
Ion transport
105.P−type ATPase
Ion transport
106.Vacuolar iron transporter 1.1
Ion transport
107.P−type ATPase
Ion transport
108.Ion transport domain

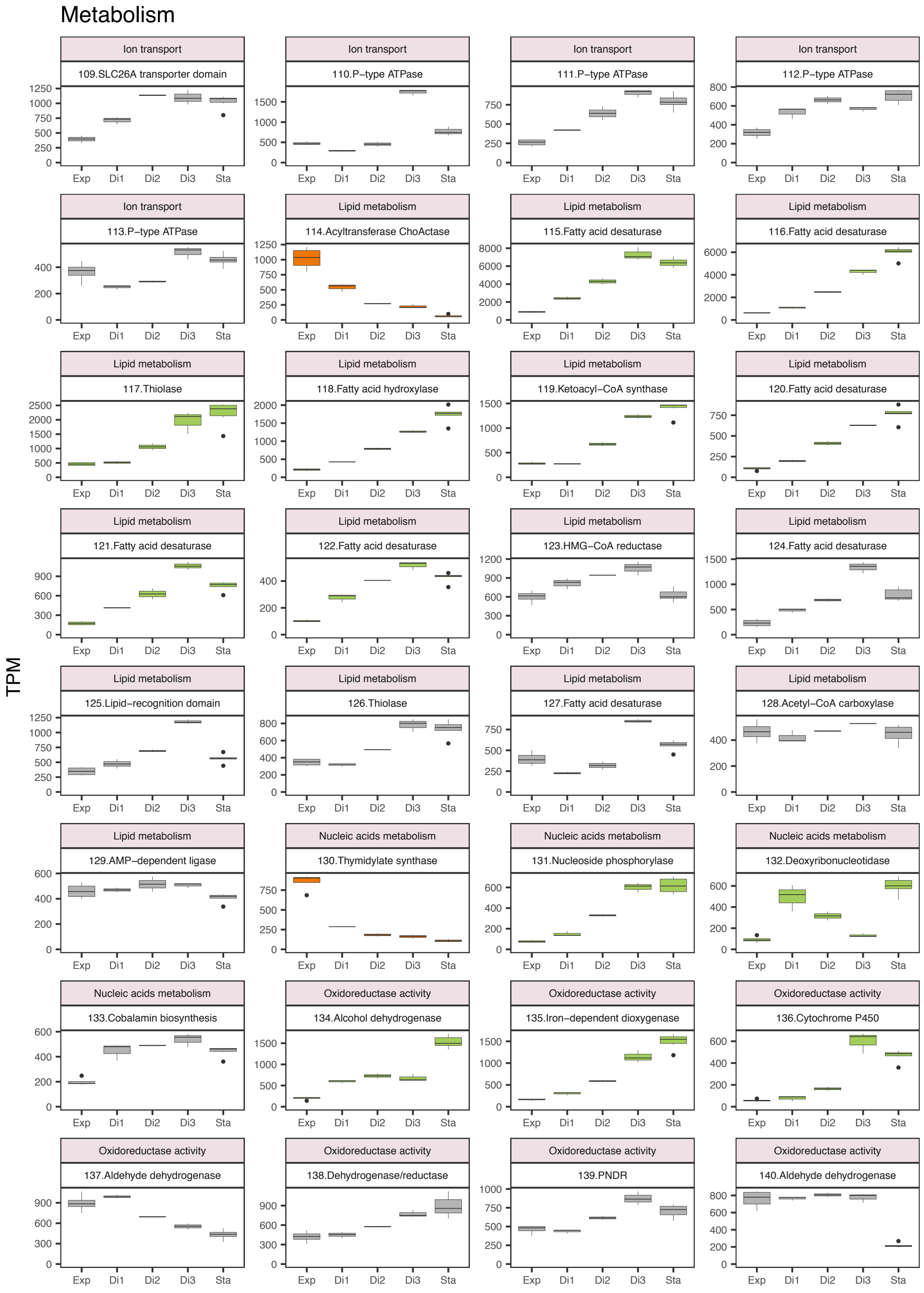
Metabolism
TPM
Ion transport
109.SLC26A transporter domain
110.P−type ATPase
111.P−type ATPase
112.P−type ATPase
113.P−type ATPase
Lipid metabolism
114.Acyltransferase ChoActase
115.Fatty acid desaturase
116.Fatty acid desaturase
117.Thiolase
118.Fatty acid hydroxylase
119.Ketoacyl−CoA synthase
120.Fatty acid desaturase
121.Fatty acid desaturase
122.Fatty acid desaturase
123.HMG−CoA reductase
124.Fatty acid desaturase
125.Lipid−recognition domain
126.Thiolase
127.Fatty acid desaturase
128.Acetyl−CoA carboxylase
129.AMP−dependent ligase
Nucleic acids metabolism
130.Thymidylate synthase
131.Nucleoside phosphorylase
132.Deoxyribonucleotidase
133.Cobalamin biosynthesis
Oxidoreductase activity
134.Alcohol dehydrogenase
135.Iron−dependent dioxygenase
136.Cytochrome P450
137.Aldehyde dehydrogenase
138.Dehydrogenase/reductase
139.PNDR
140.Aldehyde dehydrogenase
Exp
Di1
Di2
Di3
Sta

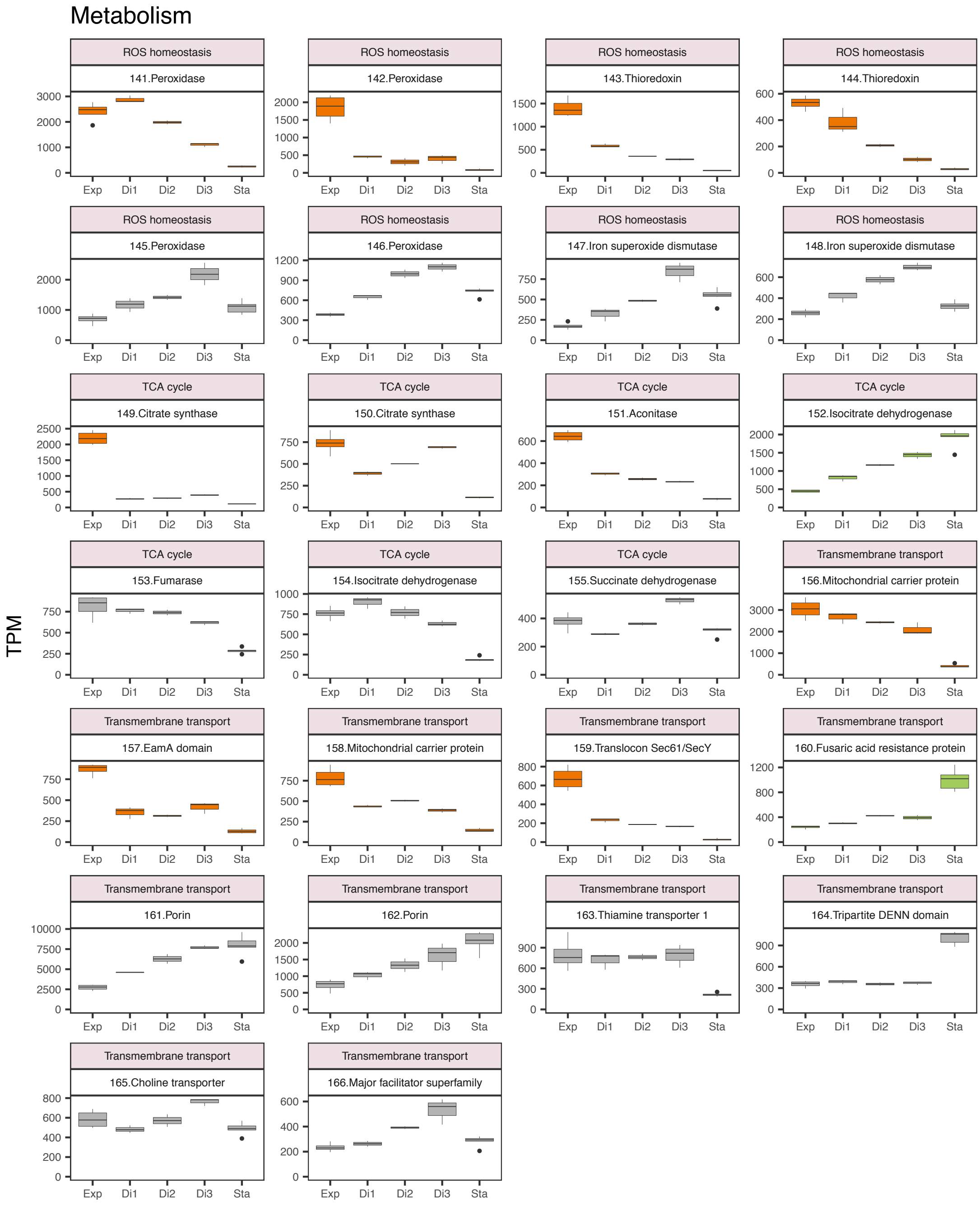
Metabolism
TPM
ROS homeostasis
141.Peroxidase
142.Peroxidase
143.Thioredoxin
144.Thioredoxin
145.Peroxidase
146.Peroxidase
147.Iron superoxide dismutase
148.Iron superoxide dismutase
TCA cycle
149.Citrate synthase
150.Citrate synthase
151.Aconitase
152.Isocitrate dehydrogenase
153.Fumarase
154.Isocitrate dehydrogenase
155.Succinate dehydrogenase
Transmembrane transport
156.Mitochondrial carrier protein
157.EamA domain
158.Mitochondrial carrier protein
159.Translocon Sec61/SecY
160.Fusaric acid resistance protein
161.Porin
162.Porin
163.Thiamine transporter 1
164.Tripartite DENN domain
165.Choline transporter
166.Major facilitator superfamily
Exp
Di1
Di2
Di3
Sta

Protein cellular processes

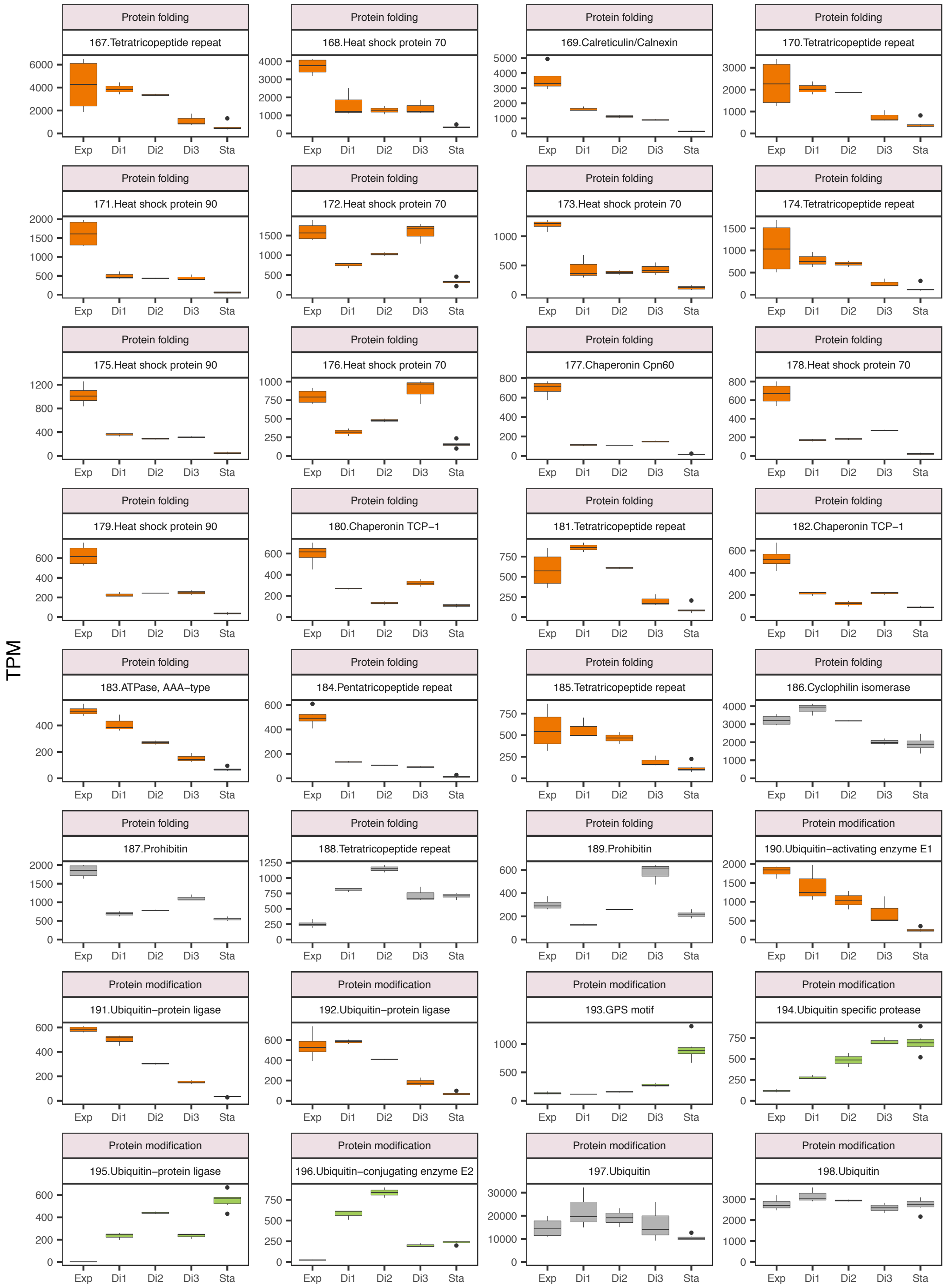

Protein cellular processes

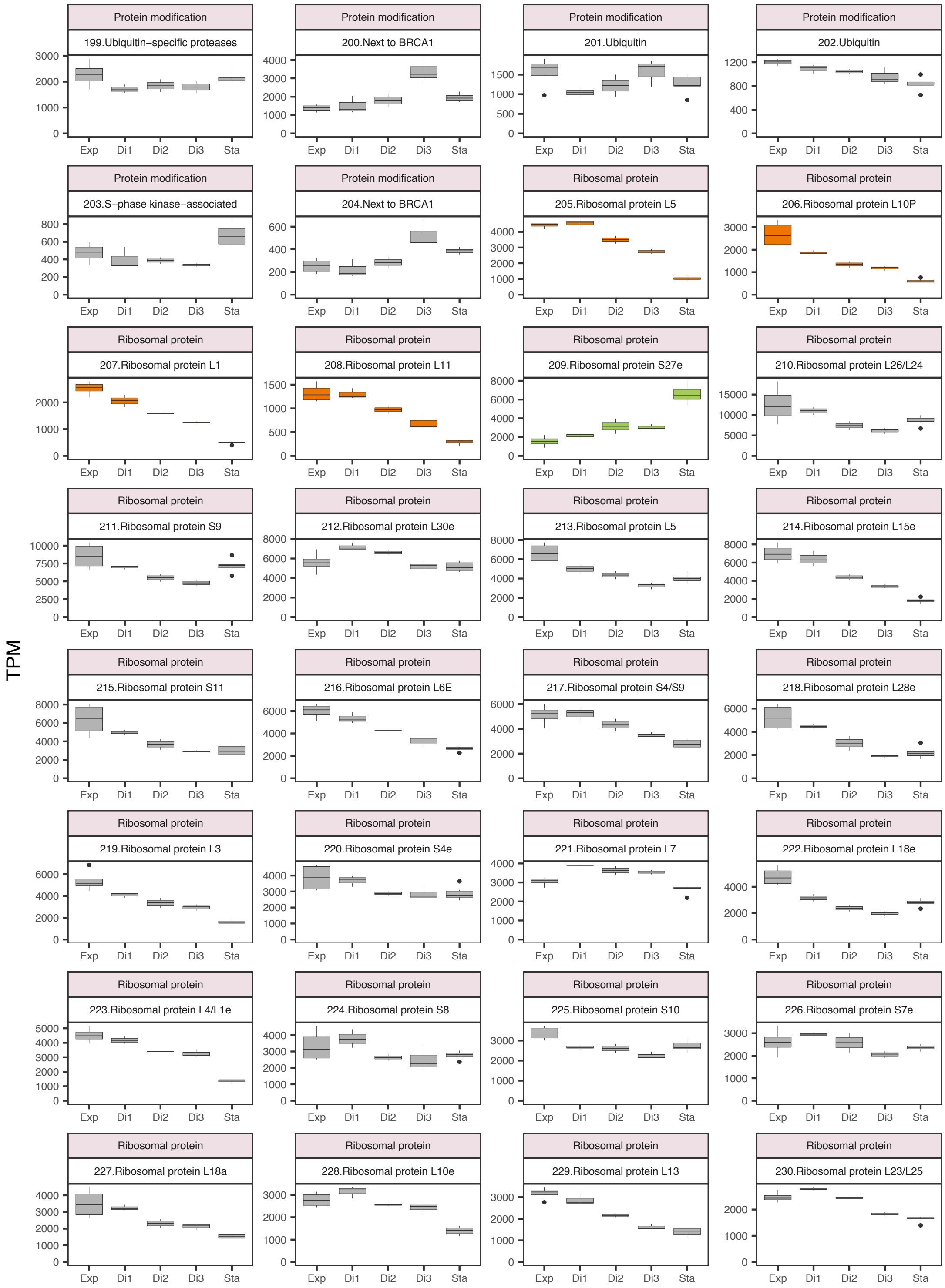

Protein cellular processes

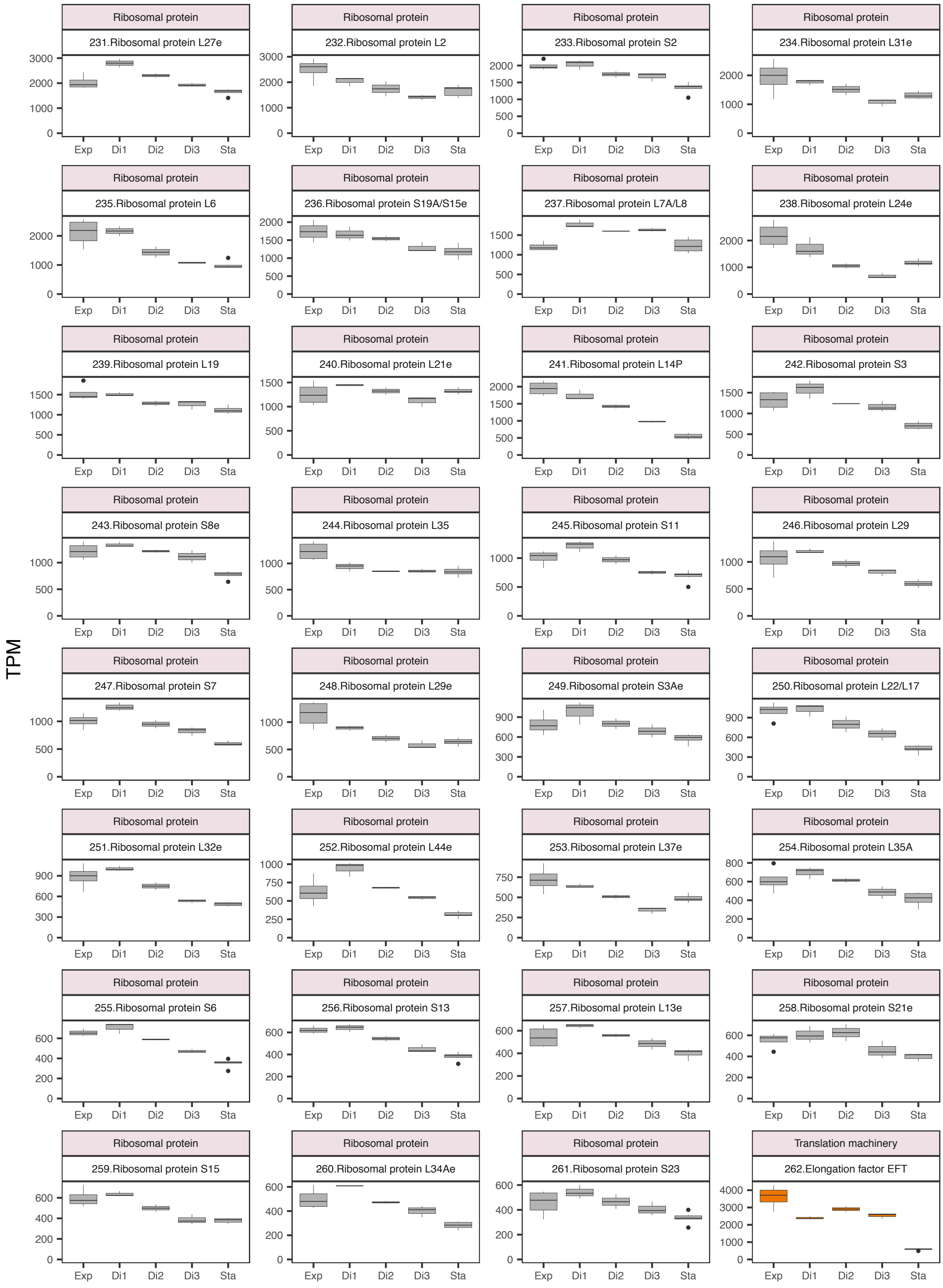

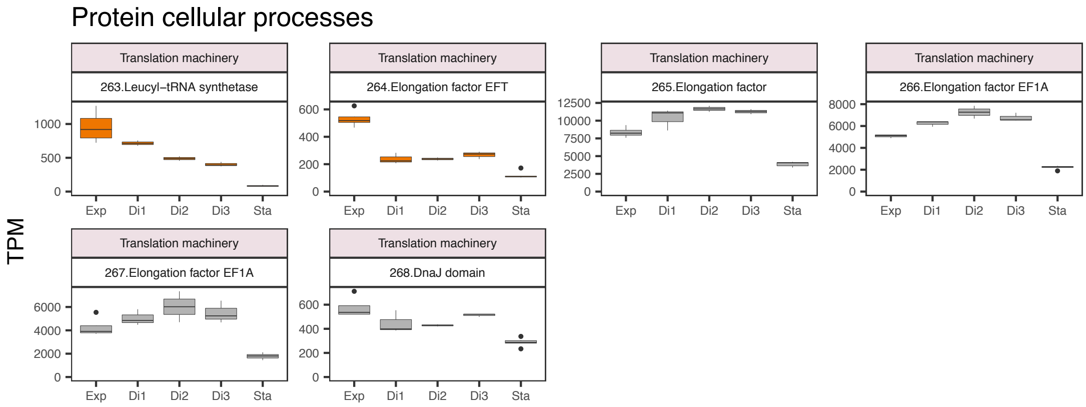

Protein cellular processes
TPM
Translation machinery
263.Leucyl−tRNA synthetase
264.Elongation factor EFT
265.Elongation factor
266.Elongation factor EF1A
267.Elongation factor EF1A
268.DnaJ domain
Exp
Di1
Di2
Di3
Sta

Signaling and cell response

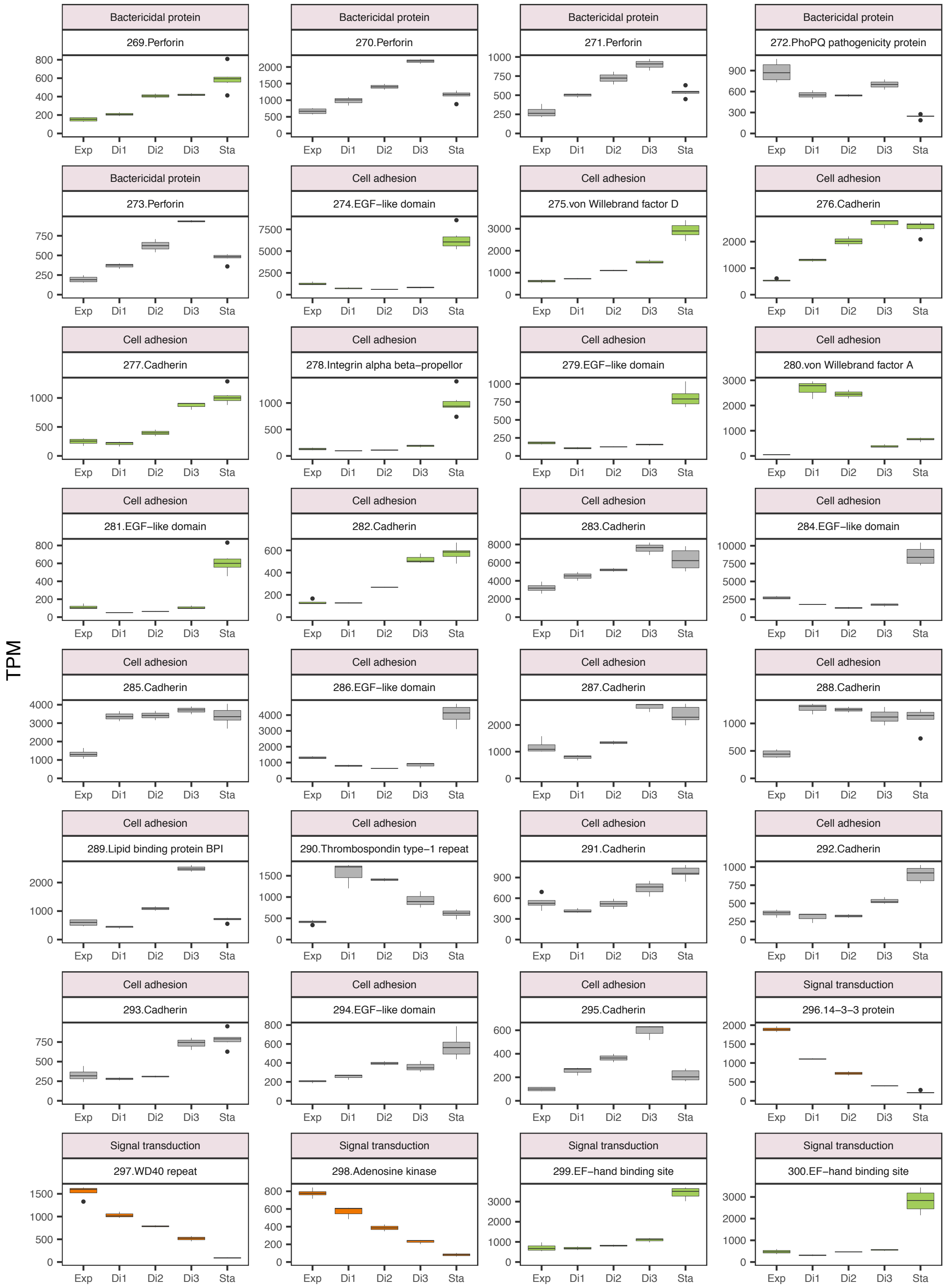

Signaling and cell response

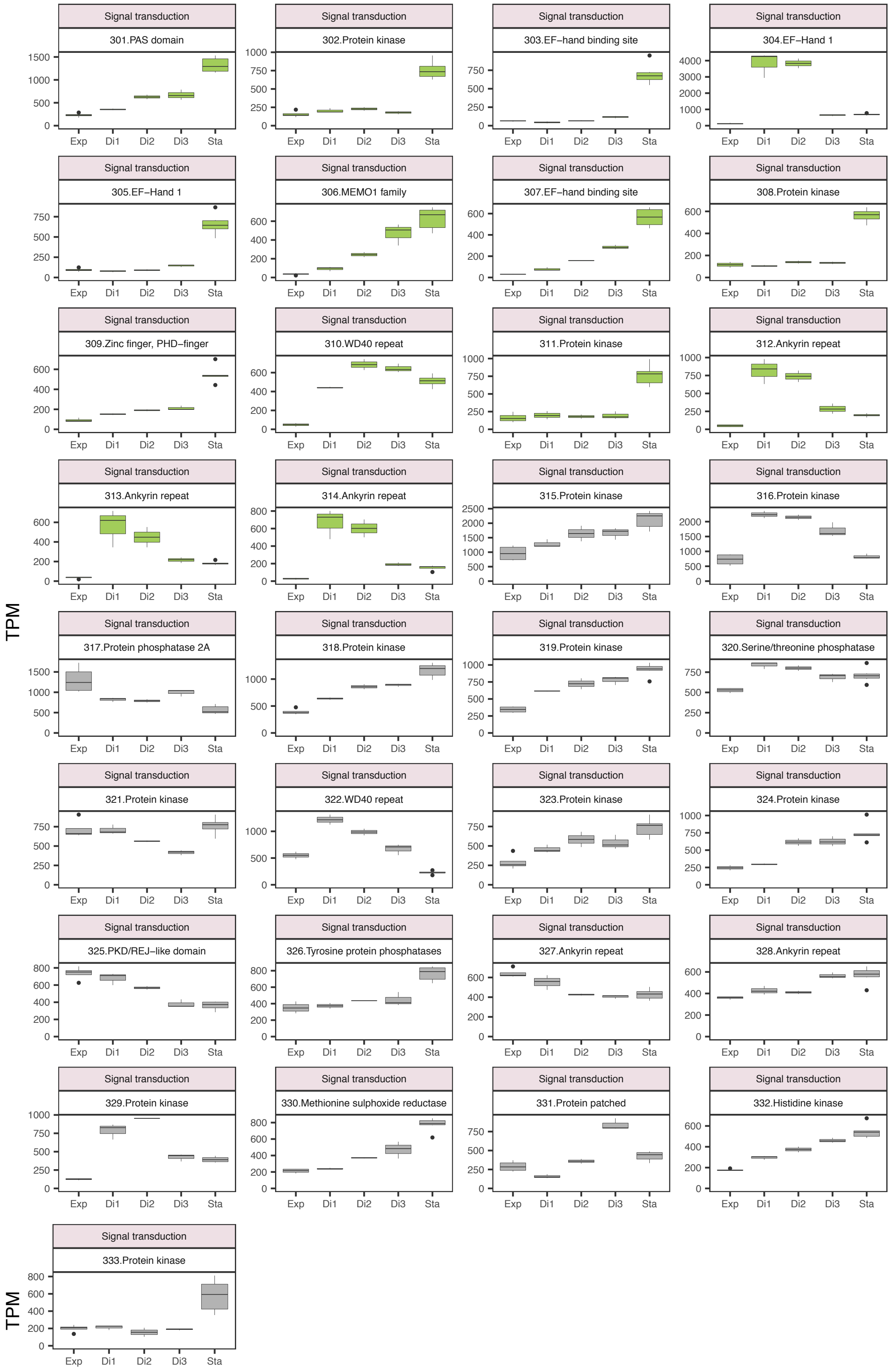

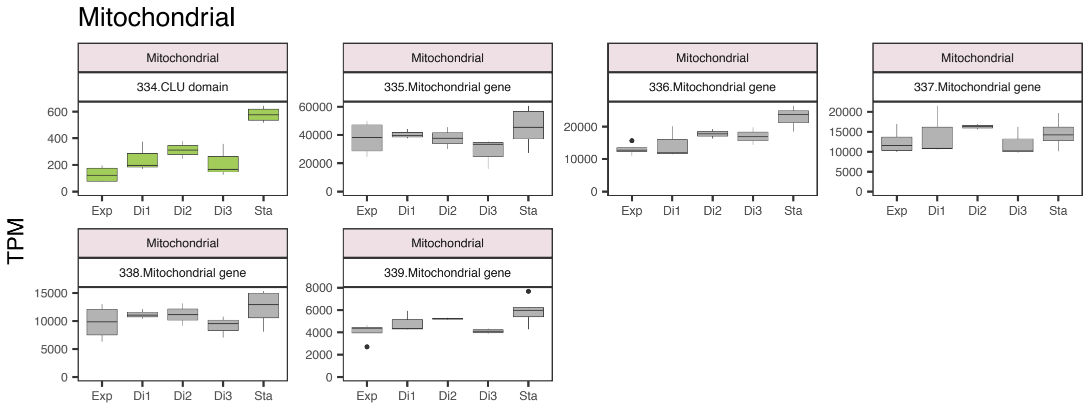
Mitochondrial
TPM
Mitochondrial
334.CLU domain
335.Mitochondrial gene
336.Mitochondrial gene
337.Mitochondrial gene
338.Mitochondrial gene
339.Mitochondrial gene
Exp
Di1
Di2
Di3
Sta

Unknown

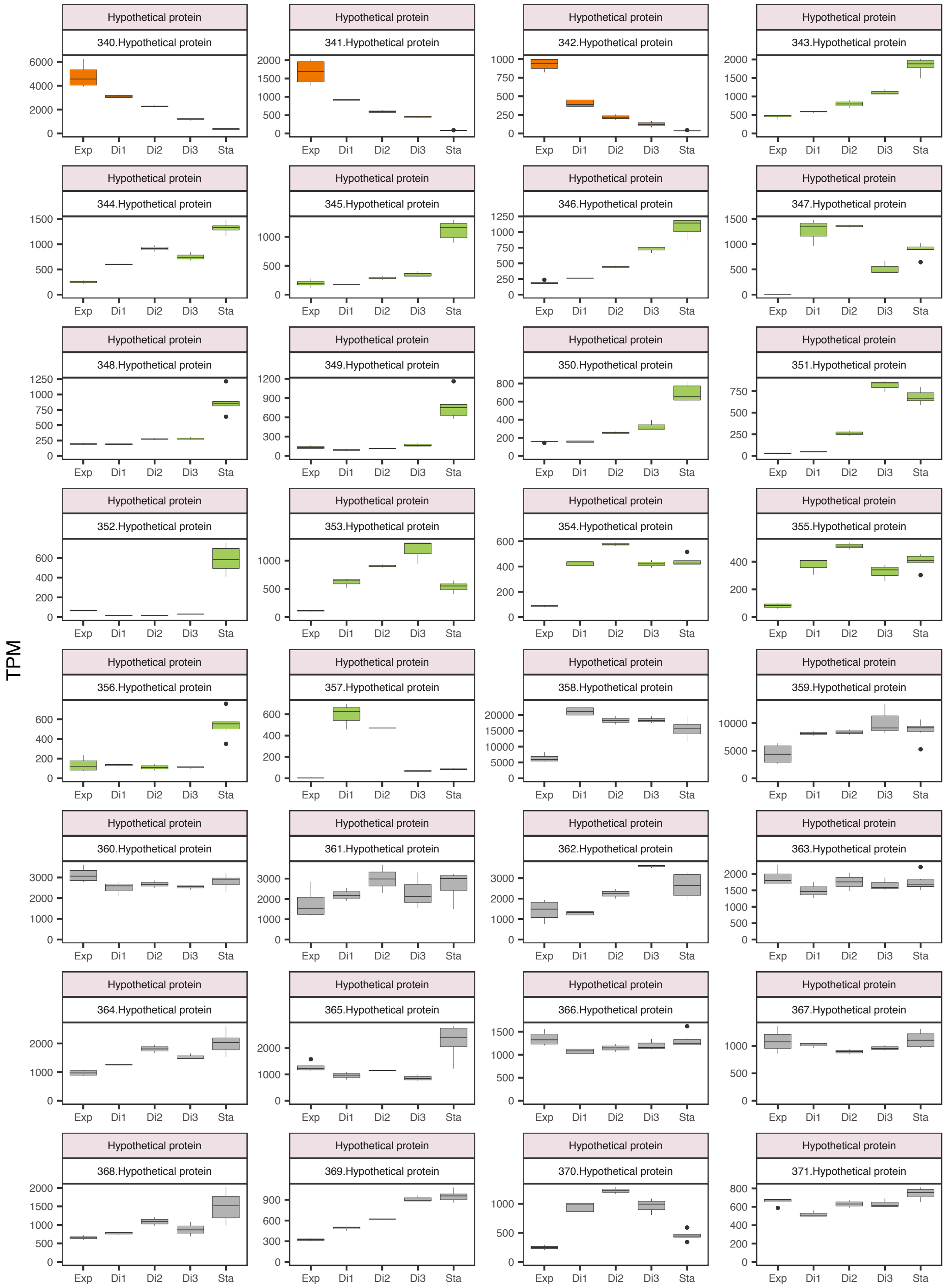
TPM
Hypothetical protein
340.Hypothetical protein
341.Hypothetical protein
342.Hypothetical protein
343.Hypothetical protein
344.Hypothetical protein
345.Hypothetical protein
346.Hypothetical protein
347.Hypothetical protein
348.Hypothetical protein
349.Hypothetical protein
350.Hypothetical protein
351.Hypothetical protein
352.Hypothetical protein
353.Hypothetical protein
354.Hypothetical protein
355.Hypothetical protein
356.Hypothetical protein
357.Hypothetical protein
358.Hypothetical protein
359.Hypothetical protein
360.Hypothetical protein
361.Hypothetical protein
362.Hypothetical protein
363.Hypothetical protein
364.Hypothetical protein
365.Hypothetical protein
366.Hypothetical protein
367.Hypothetical protein
368.Hypothetical protein
369.Hypothetical protein
370.Hypothetical protein
371.Hypothetical protein
Exp
Di1
Di2
Di3
Sta

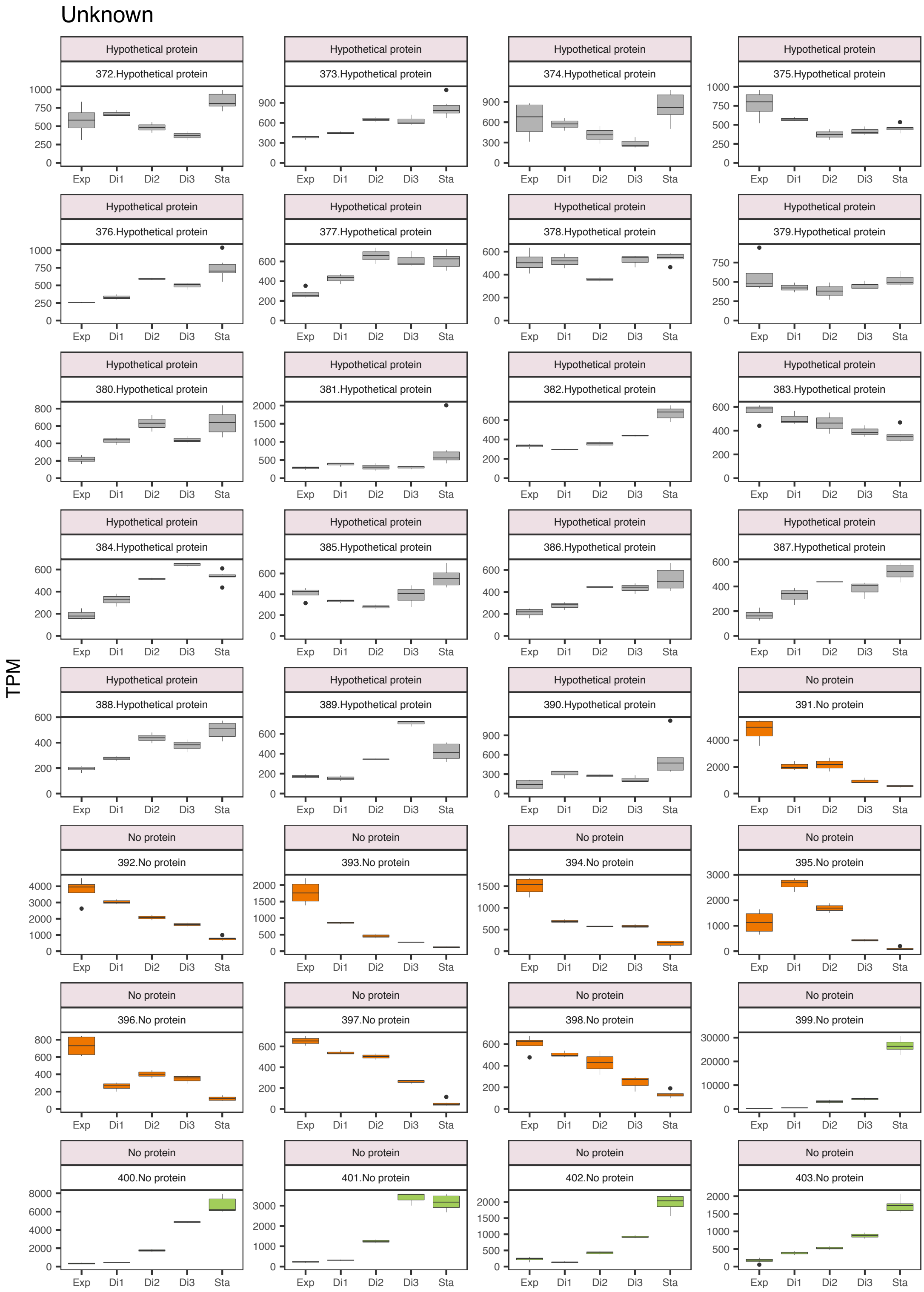
Unknown
TPM
Hypothetical protein
372.Hypothetical protein
373.Hypothetical protein
374.Hypothetical protein
375.Hypothetical protein
376.Hypothetical protein
377.Hypothetical protein
378.Hypothetical protein
379.Hypothetical protein
380.Hypothetical protein
381.Hypothetical protein
382.Hypothetical protein
383.Hypothetical protein
384.Hypothetical protein
385.Hypothetical protein
386.Hypothetical protein
387.Hypothetical protein
388.Hypothetical protein
389.Hypothetical protein
390.Hypothetical protein
No protein
391.No protein
392.No protein
393.No protein
394.No protein
395.No protein
396.No protein
397.No protein
398.No protein
399.No protein
400.No protein
401.No protein
402.No protein
403.No protein
Exp
Di1
Di2
Di3
Sta

Unknown

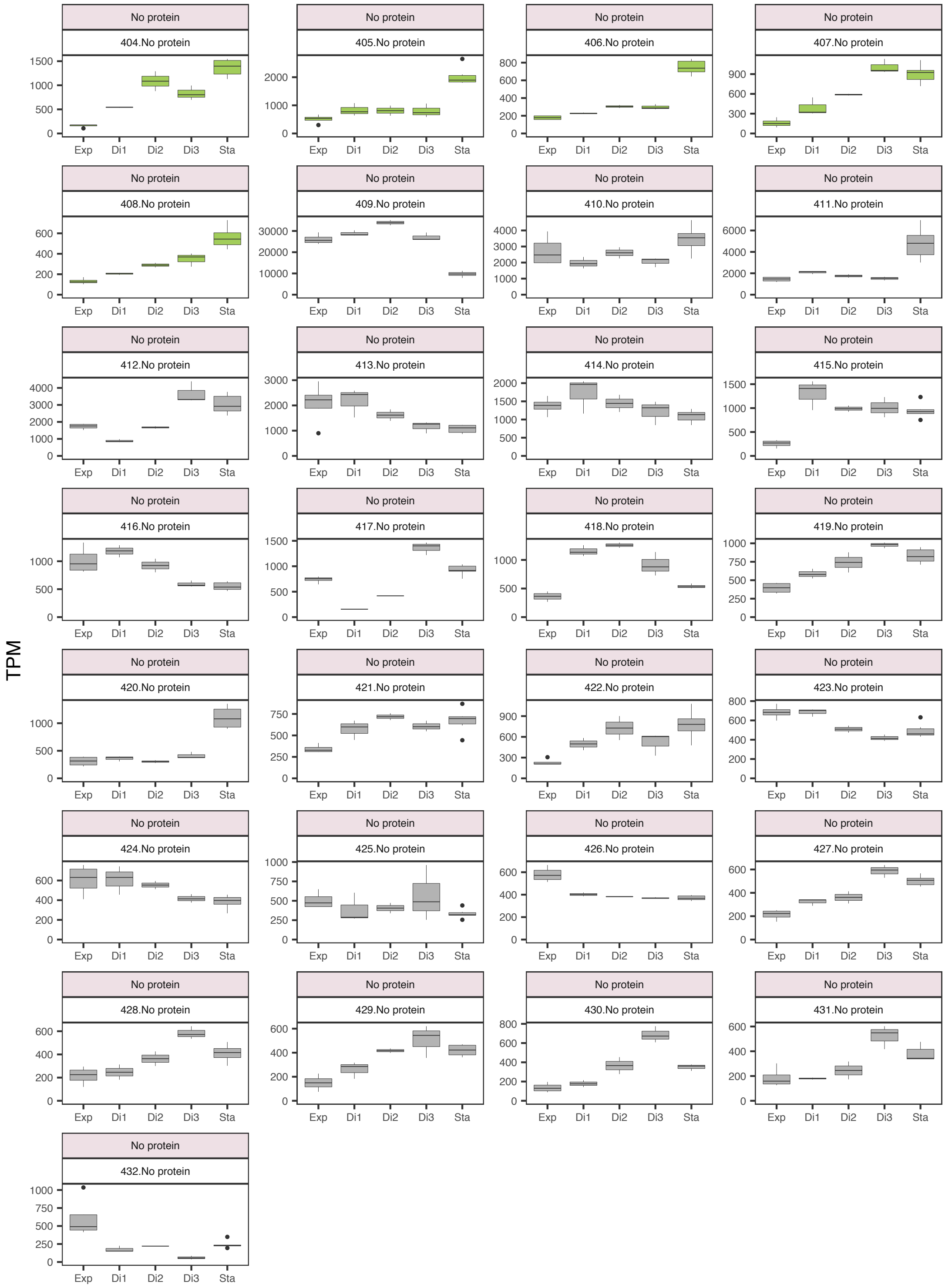
TPM
No protein
404.No protein
405.No protein
406.No protein
407.No protein
408.No protein
409.No protein
410.No protein
411.No protein
412.No protein
413.No protein
414.No protein
415.No protein
416.No protein
417.No protein
418.No protein
419.No protein
420.No protein
421.No protein
422.No protein
423.No protein
424.No protein
425.No protein
426.No protein
427.No protein
428.No protein
429.No protein
430.No protein
431.No protein
432.No protein
Exp
Di1
Di2
Di3
Sta

Table S1 Naming of samples, indicating the phase, the biological and technical replicate, and number of reads at different steps: raw reads, reads removed (after quality control or because they affiliate with the bacterial genome or the rDNA operon), clean reads, and reads finally mapping to the *C. burkhardae de novo* transcriptome

				Million reads (forward + reverese)					
Name	Phase	Bottle	Replicate	Raw reads	Reads removed by Trimming	Reads removed by MED134	Reads removed by rDNA	Clean reads	Mapped reads
Exp-B-a	Exponential	B	a	16.4	4.6	4.8	2.4	4.6	**3.5**
Exp-B-b	Exponential	B	b	15.1	4.8	3.9	2.6	3.8	**2.9**
Exp-C-a	Exponential	C	a	9.7	2.3	3.5	1.5	2.4	**1.5**
Exp-C-b	Exponential	C	b	15.9	4.7	5.0	2.5	3.7	**2.7**
Di1-A	Dilution-1	A	-	11.5	2.4	1.9	2.4	4.8	**3.5**
Di1-B	Dilution-1	B	-	19.6	6.9	2.2	3.1	7.2	**5.3**
Di1-C	Dilution-1	C	-	12.9	4.4	1.5	2.1	5.0	**3.6**
Di2-A	Dilution-2	A	-	18.1	5.8	1.7	5.1	5.5	**3.7**
Di2-B	Dilution-2	B	-	15.3	4.0	1.5	4.4	5.4	**3.6**
Di3-A	Dilution-3	A	-	14.3	5.2	0.9	3.2	4.9	**3.0**
Di3-B	Dilution-3	B	-	11.4	3.7	1.0	2.1	4.6	**3.3**
Di3-C	Dilution-3	C	-	11.3	2.8	0.8	3.4	4.3	**3.2**
Sta-A-a	Stationary	A	a	18.3	5.7	1.1	5.6	6.0	**4.7**
Sta-A-b	Stationary	A	b	16.1	5.3	0.8	4.4	5.7	**4.6**
Sta-B-a	Stationary	B	a	8.8	2.1	0.5	2.7	3.4	**2.8**
Sta-B-b	Stationary	B	b	14.2	4.5	0.7	4.1	5.0	**3.9**
Sta-C-a	Stationary	C	a	12.0	3.9	0.6	3.0	4.5	**3.6**
Sta-C-b	Stationary	C	b	21.5	6.9	1.0	3.6	9.9	**8.0**
Ino-a	Inoculum	-	a	11.8	3.9	0.5	3.4	4.0	**3.1**
Ino-b	Inoculum	-	b	15.7	5.1	0.5	4.8	5.3	**4.1**
Ino-c	Inoculum	-	c	6.0	2.5	0.2	1.5	1.8	**1.4**
			Average	14.1	4.4	1.7	3.2	4.9	**3.6**
			Max	21.5	6.9	5.0	5.6	9.9	**8.0**
			Min	6.0	2.1	0.2	1.5	1.8	**1.4**

Table S2 Species used to build the stramenopile-oriented PLAZA genome database. For species marked by an asterisk, reference GO annotation was retrieved from the GO website.

Species	Source	Publication (Pubmed ID)
Albugo candida	NCBI	21995639
Aplanochytrium kerguelense PBS07	JGI	/
Arabidopsis thaliana *	TAIR10	11130711
Aurantiochytrium limacinum	JGI	/
Aureococcus anophagefferens	JGI 1.0	21368207
Bigelowiella natans	JGI	16760254
Blastocystis hominis	NCBI	/
Cafeteria burkhardae	NCBI	31978633
Chlamydomonas reinhardtii	JGI 5.5 (Phytozome 10.2)	17932292
Dictyostelium discoideum *	ENSEMBL protist release 28	15875012
Drosophila melanogaster *	ENSEMBL release 81	10731132
Ectocarpus siliculosus	UGent ORCAE	27870061
Hyphochytrium catenoides	NCBI	29321239
MAST-1D	Co-assembly from single cells	/
MAST-11	Co-assembly from single cells	/
MAST-3A	Co-assembly from single cells	/
MAST-3F	Co-assembly from single cells	/
MAST-4A	Co-assembly from single cells	/
MAST-4C	Co-assembly from single cells	/
MAST-4E	Co-assembly from single cells	/
MAST-7	Co-assembly from single cells	/
MAST-9	Co-assembly from single cells	/
Nannochloropsis gaditana	ENSEMBL protist release 28	23966634
Phaeodactylum tricornutum	ENSEMBL protist release 28	18923393
Phytophthora sojae	ENSEMBL protist release 28	16946065
Pseudo-nitzschia multiseries	JGI 1.0	/
Pythium ultimum	NCBI	20626842
Saccharomyces cerevisiae strain S288C *	ENSEMBL release 81	8849441
Saprolegnia parasitica	NCBI	23785293
Schizochytrium aggregatum ATCC 28209	JGI	/
Schizosaccharomyces pombe *	ENSEMBL fungi release 28	11859360
Thalassiosira pseudonana	JGI	15459382

Table S3 Growth properties of *C. burkhardae* growing on *Dokdonia* MED134 in the three batch cultures established.

	**Bottle A**	**Bottle B**	**Bottle C**
Growth rate (h^{-1})	0.164	0.152	0.154
Doubling time (h)	4.2	4.6	4.5
Maximal abundance (10^4 cells ml^{-1})	7.5	8.6	8.1
Grazing rate (bacteria $flagellate^{-1}$ h^{-1})	49.3	41.6	40.4
Growth Efficiency (%)	35.9	39.5	41.1
Decay rate in stationary (h^{-1})	0.005	0.006	0.005
Half-time decay in stationary (h)	140.4	120.7	129.5

Table S4 Number of differentially expressed genes in pairwise comparisons among the five phases. Each line indicates the number of upregulated genes of the phase labeled per line (against the phase labeled per columns).

		as compared with				
		Exponential	***Dilution 1***	***Dilution 2***	***Dilution 3***	***Stationary***
**Upregulated in**	**Exponential**	-	43	235	547	1231
	**Dilution 1**	195	-	34	366	785
	**Dilution 2**	368	34	-	50	289
	**Dilution 3**	445	118	18	-	224
	**Stationary**	825	419	182	245	-
